# Deregulated immune cell recruitment orchestrated by c-MET impairs pulmonary inflammation and fibrosis

**DOI:** 10.1186/s12931-024-02884-1

**Published:** 2024-06-22

**Authors:** Catarina Barbosa-Matos, Caroline Borges-Pereira, Sofia Libório-Ramos, Raquel Fernandes, Marcela Oliveira, Ana Mendes-Frias, Ricardo Silvestre, Nuno S. Osório, Hélder N. Bastos, Rita F. Santos, Susana Guimarães, António Morais, Massimiliano Mazzone, Agostinho Carvalho, Cristina Cunha, Sandra Costa

**Affiliations:** 1 Life and Health Sciences Research Institute (ICVS), School of Medicine, University of Minho, Campus Gualtar, Braga, 4710-057 Portugal; 2 ICVS/3B’s - PT Government Associate Laboratory, Braga/Guimarães, Portugal; 3 Department of Pneumology, Centro Hospitalar do São João, Porto, Portugal; 4 i3S - Instituto de Investigação e Inovação em Saúde, University of Porto, Porto, Portugal; 5 Faculty of Medicine, University of Porto, Porto, Portugal; 6 School of Health Sciences – Polytechnic of Porto, Porto, Portugal; 7 Department of Pathology, Centro Hospitalar Universitário de São João, Porto, Portugal; 8 Laboratory of Tumor Inflammation and Angiogenesis, Center for Cancer Biology, VIB, Louvain, Belgium; 9 Laboratory of Tumor Inflammation and Angiogenesis, Department of Oncology, KU Leuven, Louvain, Belgium

**Keywords:** Bleomycin, c-MET, ILDs, Inflammation, Pulmonary fibrosis

## Abstract

**Background:**

Pulmonary fibrosis (PF) represents the pathologic end stage of several interstitial lung diseases (ILDs) associated with high morbidity and mortality rates. However, current treatments can only delay disease progression rather than provide a cure. The role of inflammation in PF progression is well-established, but new insights into immune regulation are fundamental for developing more efficient therapies. c-MET signaling has been implicated in the migratory capacity and effector functions of immune cells. Nevertheless, the role of this signaling pathway in the context of PF-associated lung diseases remains unexplored.

**Methods:**

To determine the influence of c-MET in immune cells in the progression of pulmonary fibrosis, we used a conditional deletion of *c-Met* in immune cells. To induce pulmonary fibrosis mice were administered with bleomycin (BLM) intratracheally. Over the course of 21 days, mice were assessed for weight change, and after euthanasia at different timepoints, bronchoalveolar lavage fluid cells and lung tissue were assessed for inflammation and fibrosis. Furthermore, c-MET expression was assessed in cryobiopsy sections, bronchoalveolar lavage fluid cells samples and single cell RNA-sequencing dataset from human patients with distinct interstitial lung diseases.

**Results:**

c-MET expression was induced in lung immune cells, specifically in T cells, interstitial macrophages, and neutrophils, during the inflammatory phase of BLM-induced PF mouse model. Deletion of *c-Met* in immune cells correlated with earlier weight recovery and improved survival of BLM-treated mice. Moreover, the deletion of *c-Met* in immune cells was associated with early recruitment of the immune cell populations, normally found to express c-MET, leading to a subsequent attenuation of the cytotoxic and proinflammatory environment. Consequently, the less extensive inflammatory response, possibly coupled with tissue repair, culminated in less exacerbated fibrotic lesions. Furthermore, c-MET expression was up-regulated in lung T cells from patients with fibrosing ILD, suggesting a potential involvement of c-MET in the development of fibrosing disease.

**Conclusions:**

These results highlight the critical contribution of c-MET signaling in immune cells to their enhanced uncontrolled recruitment and activation toward a proinflammatory and profibrotic phenotype, leading to the exacerbation of lung injury and consequent development of fibrosis.

**Supplementary Information:**

The online version contains supplementary material available at 10.1186/s12931-024-02884-1.

## Introduction

Pulmonary fibrosis is a common denominator in several ILDs, including idiopathic pulmonary fibrosis, and can be triggered by other diseases, such as COVID-19 [1, 2]. In addition to IPF, other ILDs can develop fibrotic condition, namely patients with hypersensitivity pneumonitis (HP) and connective tissue disease-associated interstitial lung disease (CTD-ILD) [1]. HP is caused by exposure and sensitization to an inhaled antigen and can range from a purely inflammatory disease to a progressive fibrotic disease resembling IPF [3]. CTD are systemic autoimmune disorders caused by excessive immune activated inflammation that mediates organ damage [4, 5]. ILDs occur in approximately 40–50% of patients with CTD and is the main cause of morbidity and mortality [6]. IPF is the most severe form of PF and, due to the irreversibility of PF, patients present a poor prognosis and a median survival of only 2–4 years after diagnosis [7]. The complex etiology and unclear pathogenesis of IPF result in limited and inefficient therapeutic strategies [8–11]. A better understanding of the pathogenesis of IPF is therefore essential to identify new therapeutic targets.

Although the precise trigger-associated mechanisms remain unclear, under certain circumstances, chronic or repetitive injuries can evolve to fibrosis. PF is characterized by a persistent wound repair environment, resulting in the accumulation of extracellular matrix proteins such as fibronectin and collagens in the lung, irreversible tissue damage, and loss of pulmonary function [12–15]. Wound healing processes can be modulated by several factors, including the recruitment and activation of inflammatory cells. A slight imbalance in these mediators can lead to uncontrolled tissue remodeling and, ultimately, fibrosis. In IPF, the inflammatory response is characterized by excessive amounts of T helper (Th) 2 cytokines, such as interleukin (IL)-4 and IL-13 that are critical mediators of fibrotic lung remodeling [16–19]. Likewise, studies in mouse models of PF suggest that Th2 cytokines play a pro-fibrotic role [20].

The role of myeloid cells has long been recognized in lung development, homeostasis, and in the pathogenesis of PF [14]. Macrophage populations in the lung are crucial for maintaining homeostasis responding appropriately to insults [21, 22]. Alveolar macrophages are localized in the alveolar space, contributing to airway homeostasis, pulmonary defense, repair, surfactant processing, and inflammatory responses [23]. In contrast, interstitial macrophages (IMs) are present in the interstitial area of the lung, maintaining immune homeostasis and modulating tolerance toward non-threatening antigens [24, 25]. Macrophages can also modulate the inflammatory recruitment and activate myofibroblasts through the secretion of chemokines and profibrotic cytokines, respectively [13, 26, 27]. Furthermore, the phenotypic activation of macrophages dictates how they modulate fibroproliferative responses. The M1, classically activated macrophages, maintain tissue inflammation, while M2, alternatively activated macrophages, play a role in resolving lung inflammation and the aberrant wound healing cascade during fibrosis [28–31]. Neutrophils are also an important cell population of the innate immune system, which migrate to the sites of infection or tissue injury and exhibit a wide variety of effector functions such as phagocytosis, production of reactive oxygen species, degranulation, and release of neutrophil extracellular traps [32]. Despite their efficient, protective functions, the inflammatory response of neutrophils can be a double-edged sword, as it can also cause severe tissue damage. During PF, neutrophil accumulation appears to amplify uncontrolled tissue repair and fibrosis by secreting elastase, which stimulates proliferation and differentiation of fibroblasts and extracellular matrix production [33, 34]. Furthermore, neutrophil recruitment is a predictor of early mortality in patients with IPF [35].

Recently, the signaling pathway involving hepatocyte growth factor (HGF) and its tyrosine kinase receptor, c-MET, has been described in the modulation of the migratory and functional activity of immune cells. In the tumoral context, c-MET signaling is induced in neutrophils upon inflammatory stimuli, increasing their migratory capacity and reinforcing their nitric oxide release, thereby promoting cancer cell death [36]. Moreover, c-MET inhibition decreases the recruitment of neutrophils into the T cell–inflamed tumor microenvironment and draining lymph nodes in response to cytotoxic immunotherapies [37]. Also, tumoral cytotoxic T cells that express c-MET have enhanced cytolytic capacities [38]. In *Leishmania mexicana* infection, c-MET expression is activated in neutrophils and favors their recruitment to the site of infection [39]. Furthermore, in a murine model of intestinal inflammation, c-MET expression is induced mainly in neutrophils and its deletion in neutrophils reduces disease severity [40].

Although inflammation plays a critical role in the progression of PF, the role of c-MET signaling in immune cells remains to be determined. Herein, we demonstrate that c-MET expression is induced in lung immune cells in response to bleomycin (BLM) administration. Moreover, conditional deletion of *c-Met* in immune cells leads to their early recruitment to the lung but, paradoxically, to a less proinflammatory environment. This prevents subsequent uncontrolled cytotoxicity and inflammation, while promoting tissue repair, and attenuates the long-term effects and the histopathological characteristics of BLM-induced lung fibrosis. Together, these data highlight the critical contribution of c-MET signaling in immune cells to their enhanced uncontrolled recruitment and activation toward a proinflammatory and profibrotic phenotype, leading to the exacerbation of lung injury and consequent development of fibrosis. More importantly, we demonstrate that c-MET expression in T cells is increased in fibrosing ILD patients, suggesting a potential involvement of c-MET in the development of the fibrosing condition. In this sense, selective c-MET-targeted therapies might represent an attractive strategy to control tissue damage and inflammation in these patients.

## Methods

### Mice and human samples

All procedures in vivo followed the EU-adopted regulations (Directive 2010/63/EU). Ethical and regulatory approvals were consented by Direção Geral de Alimentação e Veterinária (DGAV, 003671) and conducted according to the guidelines sanctioned by the DGAV. Mice were supplied by Professor Massimiliano Mazzone, Center for Cancer Biology, VIB, KU Leuven, Belgium. Eight-week-old gender- and age-matched C57BL/6 mice were bred under specific pathogen-free condition and kept at the Life and Health Sciences Research Institute (ICVS) animal facility. Mice were fed ad libitum and kept under light/dark cycles of 12 h, temperature of 18–25 °C and humidity of 40–60%. Male, 10 to 12 weeks old, wild-type (WT, Tie2:*c-Met*^wt/wt^) and conditional knockout (cKO, Tie2:*c-Met*^fl/fl^) C57BL/6J mice were used.

This study was reviewed and approved by the Ethics Committee of Hospital de São João (CES 72–12). Transbronchial cryobiopsies and cryopreserved bronchoalveolar lavage (BAL) from ILD patients were obtained. Demographic and clinical features of studied patients are reported in Table S1 and Table S2, respectively.

### BLM-induced pulmonary fibrosis mouse model

Mice were sedated with ketamine and medetomidine and intratracheally administered with vehicle or with BLM sulphate (BML-AP302-0010, Enzo Life Sciences) as a single dose of 2.5 mg/kg in 50 µL saline.

### BAL collection and preparation of mouse lung single-cell suspensions

Briefly, mice were over anesthetized by intraperitoneal injection, then trachea was exposed by a midline incision and cannulated. Bilateral BAL recovered by a three times lavage with 1 mL of PBS. The supernatant from the BAL was frozen for the LDH and multi‐analyte flow assay. Then, animals were perfused with PBS. After dissection, the lungs were placed in dispase (734-1312, Corning) while minced into 2–3 mm^3^ pieces for 20 min. Then, 1 mg/mL DNase I (10104159001, Roche) and 5 mg/mL collagenase II (17101-015, Gibco) solution in Dulbecco's Modified Eagle Medium (21969-035, Gibco) was added to the sample. After, an enzymatic digestion period of 10 min at 37 °C, tissue disruption was performed by mechanical titrations with P1000 tip, and then a second incubation period of 10 min at 37 °C was conducted. After incubation, the disrupted tissue suspension was collected and sequentially filtered through 100 µm and 70 µm cell strainers. The supernatant was collected and stored at -80 °C for the LDH assay. BAL cells were added to the lung cell suspension, centrifuged and the pellet was resuspended in ammonium-chloride-potassium lysis buffer (A1049201, Gibco), and incubated for 4 min. Cells were then maintained in cold PBS containing 4% fetal bovine serum (10270-106, Gibco).

### Flow cytometric immunophenotyping analysis of mouse and human samples

Single-cell suspension, 1 × 10^6^ cells were preincubated with TruStain FcX anti-mouse CD16/32 antibody (101320, Biolegend) for 10 min at RT. After the blocking of non-specific binding of immunoglobulin to the Fc receptors, cells were stained with the membrane antibodies listed in Table S3, for 30 min on ice in the dark. After, cells were fixed by incubation in 4% paraformaldehyde (PFA) for 20 min on ice in the dark. Defrosted human BAL cells were preincubated with TruStain FcX anti-human CD16/32 antibody (422301, Biolegend) and then stained for 30 min with the membrane antibodies listed in Tables S4. Data were acquired on a BD® LSRII flow cytometer and BD® LSRFortessa™ Cell Analyzer, for mouse and human samples, respectively. FlowJo Software was used to analyze the data. Gating strategy is illustrated in Fig. S1A, B, for the identification of myeloid and lymphoid cells, respectively, in mouse lung and in Fig. S2 for human samples.

### Survival analysis

After BLM administration, animals were followed for 21 days. Animals’ weight was monitored every 1–3 days. Mice that reached the humane endpoint were sacrificed, evaluated with a score that accounts for the percentage weight loss, labored breathing, increased respiratory rate, decreased activity, hunched posture and ruffled fur.

### Histological examination

Lungs were perfused with PBS, fixed in 4% PFA for 24 h, subsequently paraffin-embedded, sectioned and stained with hematoxylin and eosin (H&E) and Masson’s trichrome. Inflammatory scoring system for mouse lung was performed according to Gori *et al.* [41], consisting of a scale from 0 to 4 (corresponding to no inflammation to severe inflammation, respectively, depending on the density of inflammatory infiltrates and on the alveolar septum thickness). The fibrotic score was determined according to the modified Aschcroft score [42]. Histological scoring system for lesions extension quantification in the lung was randomly performed by blindly score lung lobes, using a × 10 magnification. In both scores, the slides were independently evaluated by two researchers in a double-blind fashion. The average score from all fields was used to reach the inflammatory/fibrotic score of each animal. Representative images from the H&E and Masson’s trichrome stained tissue sections were acquired using the Olympus BX61 light microscope equipped with an Olympus DP-70 digital camera in a × 10 or × 20 magnification and processed by cellSens™ software.

### Measurement of lactate dehydrogenase (LDH) activity

The LDH activity in BAL and dissociated tissue supernatants was assayed using the LDH kit (K726, BioVision). Briefly, 2 µL of NADH standards and supernatant samples were transferred to a 96-well plate and 100 µL of LDH substrate mixture were added, the absorbance was measured at the wavelength of 450 nm in a microplate reader Varioskan Flash (Thermo Scientific). Then the plate was incubated for 30 min at 37 °C. LDH activity in samples was calculated based on the standard curve, according to the manufacturer instructions, and LDH activity in the samples was expressed in nmol/min (mU) *per* mL.

### RNA isolation and RT-qPCR

On day 7 after BLM administration, single-cell suspensions were frozen at -80 °C and RNA was isolated using ExtractMe total RNA kit (EM09, Blirt) according to the manufacturer instructions. On day 14 after BLM administration, the lungs were flash frozen in liquid nitrogen. For the RNA extraction, the tissue was firstly homogenized in TRIzol reagent (15596-026, Invitrogen), through mechanical disruption using microcentrifuge pestle and then needles with crescent gauge values.

First-strand cDNA was synthesized using the commercial Maxima First Strand cDNA synthesis kit (K1671, Thermo Fisher Scientific), following the manufacturer instructions. Real-time PCR amplifications were performed using commercial 2 × Maxima probe/ROX master mix (K0231, Thermo Fisher Scientific), containing a Hot Start Taq DNA Polymerase and TaqMan® gene expression premade assays from Applied Biosystem.

Real-time PCR amplifications were performed using commercial 2 × Maxima probe/ROX master mix (K0231, Thermo Fisher Scientific), containing a Hot Start Taq DNA Polymerase and TaqMan® gene expression premade assays from Applied Biosystem. The PCR probes analyzed at 7 days were *glyceraldehyde-3-phosphate dehydrogenase* (*Gapdh,* Mm99999915_g1), *insulin-like growth factor* (*Igf)-1* (Mm00439560), *transforming growth factor* (*Tgf)-β1* (Mm01178820_m1), and *connective tissue growth factor* (*Ctgf*, Mm01192933_g1). The PCR probes assessed at 14 days were *Gapdh* (Mm99999915_g1), *Collagen* (*Col) 1a1* (Mm00801666_g1), *Col1a2* (Mm00483888_m1), *Col3a1* (Mm00802300_m1), *Actin-α 2* (*acta-2,* Mm00725412_s1), *S100a4* (Mm00803372_g1), *Tgf-β1* (Mm01178820_m1), *Ctgf* (Mm01192933_g1), *Elastin* (*Eln,* Mm0051470_m1) and *Igf-1* (Mm00439560_m1). Gene expression levels were normalized to endogenous control *Gapdh* and performed accordingly to the 2^−ΔΔCT^ method [43]. Gene expression data was presented in relative expression fold-change based on 2^−ΔΔCT^ (cKO value) / average (2^−ΔΔCT^ (WT group)).

### LEGENDplex™ (multi‐analyte flow assay kit)

Cell suspensions from dissociated lung tissue were homogenized in 1 mL of PBS with protease inhibitors (cOmplete™, Mini, EDTA-free Protease Inhibitor Cocktail, 04693159001, Roche Diagnostic) and 0.05% Tween 20. The respective supernatants were analyzed with the following Mouse LEGENDplex™ panels. Mouse chemokines were analyzed using a mouse 13-plex multiplex bead-based assay panel, including CXCL1 (KC), Eotaxin (CCL11), MCP-1 (CCL2), IP-10 (CXCL10), MIP-1α (CCL3), MIP-1β (CCL4), RANTES (CCL5), TARC (CCL17), MIG (CXCL9), MIP-3α (CCL20), LIX (CXCL5), BLC (CXCL13) and MDC (CCL22), according to the manufacturer’s instructions (740451, Biolegend). Mouse proinflammatory cytokines, were evaluated using a mouse 8-plex multiplex bead-based assay panel, including IL-18, IL-23, IL-12p70, TNF-α, IL-12p40, IL-1β and IL-6, according to the manufacturer’s instructions (740848, Biolegend). Additional mouse cytokines, were quantified using a mouse 12-plex multiplex bead-based assay panel, including, IL-2, IL-4, IL-5, IL-6, IL-9, IL-10, IL-13, IL-17A, IL-17F, IL-22, IFN-γ and TNF-α, according to the manufacturer’s instructions (741044, Biolegend).

BAL supernatants were analyzed with LEGENDplex™ mouse macrophage 13-plex multiplex bead-based assay panel, including CXCL1 (KC), TGF-β1, IL-18, IL-23, MDC (CCL22), IL-10, IL-12p70, IL-6, TNF-α, G-CSF, TARC (CCL17), IL-12p40, and IL-1β, according to the manufacturer’s instructions (740852, Biolegend).

The assays were performed in a V‐bottom plate according to the manufacturer's protocol. Data acquisition was done using the BD® LSRII flow cytometer. BioLegend's LEGENDplex™ data analysis software was applied for analysis.

### Measurement of lung hydroxyproline

Hydroxyproline concentration of the lungs was measured to determine the total collagen content in the lung using a colorimetric hydroxyproline assay kit (MAK008, Sigma), according to the manufacturer’s instructions. In brief, the lungs were homogenized and then hydrolyzed in 6 N HCl at 120 °C for 3 h in a dry block heater. Hydrolyzed samples were transferred to a 96-well plate and evaporated to dryness at 70 °C. Then, 100 µL of chloramine T reagent was added to each sample and incubated at room temperature (RT) for 5 min, followed by the addition of 100 µL of 4-(Dimethylamine) benzaldehyde (DMAB) reagent to each sample and standard well and incubated at 60 °C for 90 min. Absorbance of oxidized hydroxyproline was read at 560 nm in a microplate reader Varioskan Flash (Thermo Scientific). The amount of hydroxyproline was calculated from the standard curve and its concentration was expressed in micrograms *per* milligram of lung.

### Immunofluorescence

Human lung cryobiopsy slides were incubated for 1 h at 60 °C, deparaffinized in xylene and dehydrated in 100% ethanol. Antigen retrieval was performed in 10 mM citrate buffer (pH 6.0) heated on microwaved (500 W) during 15 min. Non-specific antibody binding sites in lung tissue sections were blocked for 1 h at RT with blocking solution (BS), 1:20 horse serum in PBS. Slides were incubated at RT overnight with the primary antibody 1:200 CD3 (ab16669, Abcam), 1:50 CD68 (orb197999, Biorbyt) or 1:300 myeloperoxidase (14569, Cell Signaling Technology) diluted in BS. Slides were then stained with the secondary antibody 1:150 horse anti-rabbit IgG (BA-1100, Vector Labs) diluted in BS, for 2 h at RT. Tissue sections were further incubated with 1:100 Alexa Fluor™ 568 streptavidin (S11226, invitrogen) diluted in BS for 1 h at RT. For sequential double staining, non-specific antibody binding sites in lung tissue sections were blocked for 1 h at RT in BS, PBS with 1:5 donkey serum. Slides were incubated at 4ºC overnight with the primary antibody 1:100 c-MET (ab51067, Abcam) diluted in BS. Slides were then stained with the secondary antibody 1:150 DyLight™ 488 donkey anti-rabbit IgG (406404, Biolegend) diluted in BS for 2 h at RT. Nuclei were counterstained with 0.1 μg/mL DAPI (4′,6-diamidino-2-phenylindole) for 5 min at RT. After each mentioned incubation, slides were rinsed with PBS containing 0.02% (v/v) Tween-20. Slides were mounted with PermaFluor Aqueous Mounting Medium (Thermo Fisher) and images acquired using an Olympus BX61 light microscope equipped with an Olympus DP-70 digital camera in a × 20 magnification, processed by cellSens software.

### Single cell RNA-sequencing analysis

Data from a single cell RNA-sequencing analysis of lungs from eight donor and nine patients with various forms of pulmonary fibrosis, including IPF, HP and CTD-ILD, were used [44]. Raw data were retrieved from GEO with the identifier GSE122960, imported into the R environment (version 3.4), and analyzed with the Seurat package (http://satijalab.org/seurat/, version 2.2). R code used for data analysis is available at https://github.com/NUPulmonary/Reyfman2018.

### Statistical analysis

Data were analyzed and plotted using the GraphPad Prism 6.00 (GraphPad Software, San Diego, CA). All results were expressed as group mean ± standard error of the mean (SEM). For all comparisons, normality was assessed with Shapiro-Wilk test, if normality is not verified non-parametric tests were used. Comparisons between two groups were performed either with parametric unpaired two-tailed Student’s t-test or non-parametric two-tailed Mann–Whitney test. Comparisons between three groups were performed either with parametric one-way ANOVA or non-parametric Kruskall-Wallis test. Survival curves (Kaplan–Meier plots) were compared using the log-rank test. A *p* value of less than 0.05 was considered a statistically significant difference.

## Results

### c-MET expression is induced in lung immune cells during BLM-induced pulmonary inflammation

**Fig. 1:**
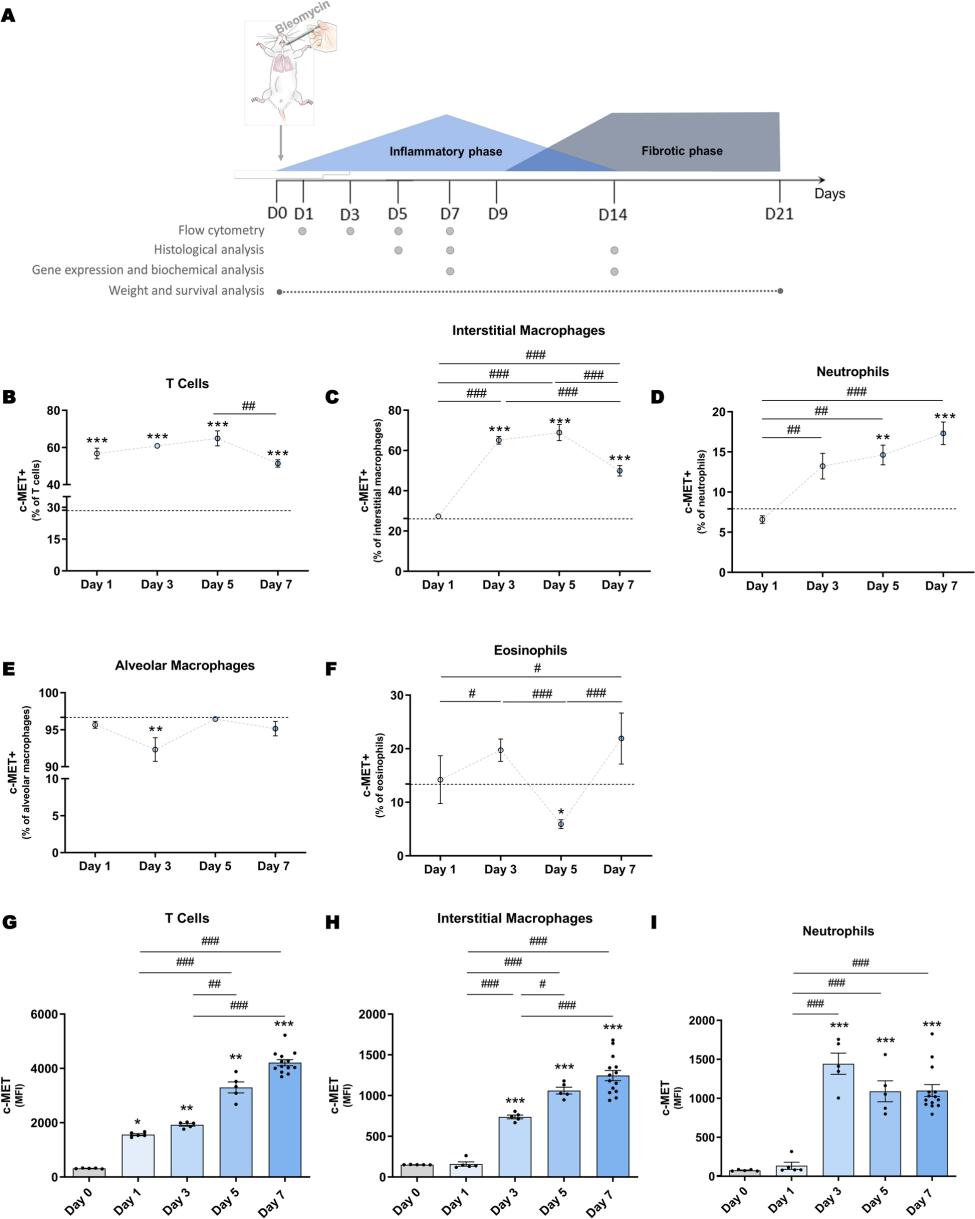
c-MET expression is enhanced in immune cells during BLM-induced lung inflammation. (**A**) Mouse model of pulmonary fibrosis, induced by BLM intratracheal administration. This model comprises an inflammatory reaction which vanishes gradually and it is followed by the fibrotic phase. Several readouts were analyzed at specific time points throughout both phases of disease progression, which are mentioned in the scheme associated with the respective time point. Percentage of c-MET^+^ among (**B**) T cells, (**C**) interstitial macrophages, (**D**) neutrophils, (**E**) alveolar macrophages and (**F**) eosinophils, horizontal dashed line represents the mean of the vehicle group (day 0), and MFI of c-MET in lung (**G**) T cells, (**H**) interstitial macrophages and (**I**) neutrophils in the lungs of vehicle-administered mice (day 0) and throughout the inflammatory phase of BLM-induced PF (day 1, day 3, day 5 and day 7). *n* = 5–14 *per* group. Data are expressed as mean ± SEM and were analyzed with one-way ANOVA with Tukey’s multiple comparisons test. *p < 0.05, **p < 0.01, ***p < 0.001, comparison with vehicle group. #*p* < 0.05, ##*p* < 0.01, ###*p* < 0.001, comparison between time points. For all the experiments, similar results were obtained in three independent experiments

Pulmonary inflammation and fibrosis were induced in mice by intratracheal administration of bleomycin (Fig. 1A). This PF mouse model is well-established, characterized by an inflammatory phase peaking on day 7, followed by the fibrotic phase peaking on day 14 [45–47].

To elucidate the potential involvement of c-MET signaling in immune cells during the inflammatory phase of the BLM-induced PF mouse model, we evaluated its expression at different time points of the inflammatory phase (Fig. 1A). Flow cytometry analysis revealed a significant increase in both the percentage and mean fluorescent intensity (MFI) of lung c-MET^+^ T cells, which represent more than 50% of the total T cells, during the inflammatory phase of the disease (Fig. 1B, G). Both the percentage and the MFI of c-MET^+^ interstitial lung macrophages (Fig. 1C, H) increased after day 3 post BLM administration, representing approximately 60% of total IMs. Both the percentage and MFI of c-MET^+^ lung neutrophils (Fig. 1D, I), began to increase from day 3 post-BLM administration, representing around 15% of the total neutrophils. No differences were found in alveolar macrophages (Fig. 1E) or in eosinophils (Fig. 1F). In addition, c-MET is known to be essential for the maturation and regeneration of epithelial cells in other models of tissue injury and regeneration, and, in fact, we also found an increase in c-MET expression in lung epithelial cells after BLM administration, whereas no expression was detected in lung endothelial cells throughout the inflammatory phase of the BLM-induced PF mouse model (Fig. S3). These results clearly demonstrate that c-MET is induced during the inflammatory phase of BLM-induced PF in immune cells.

### *c-Met* deletion in immune cells attenuates severity and lung injury of BLM-induced pulmonary fibrosis

**Fig. 2:**
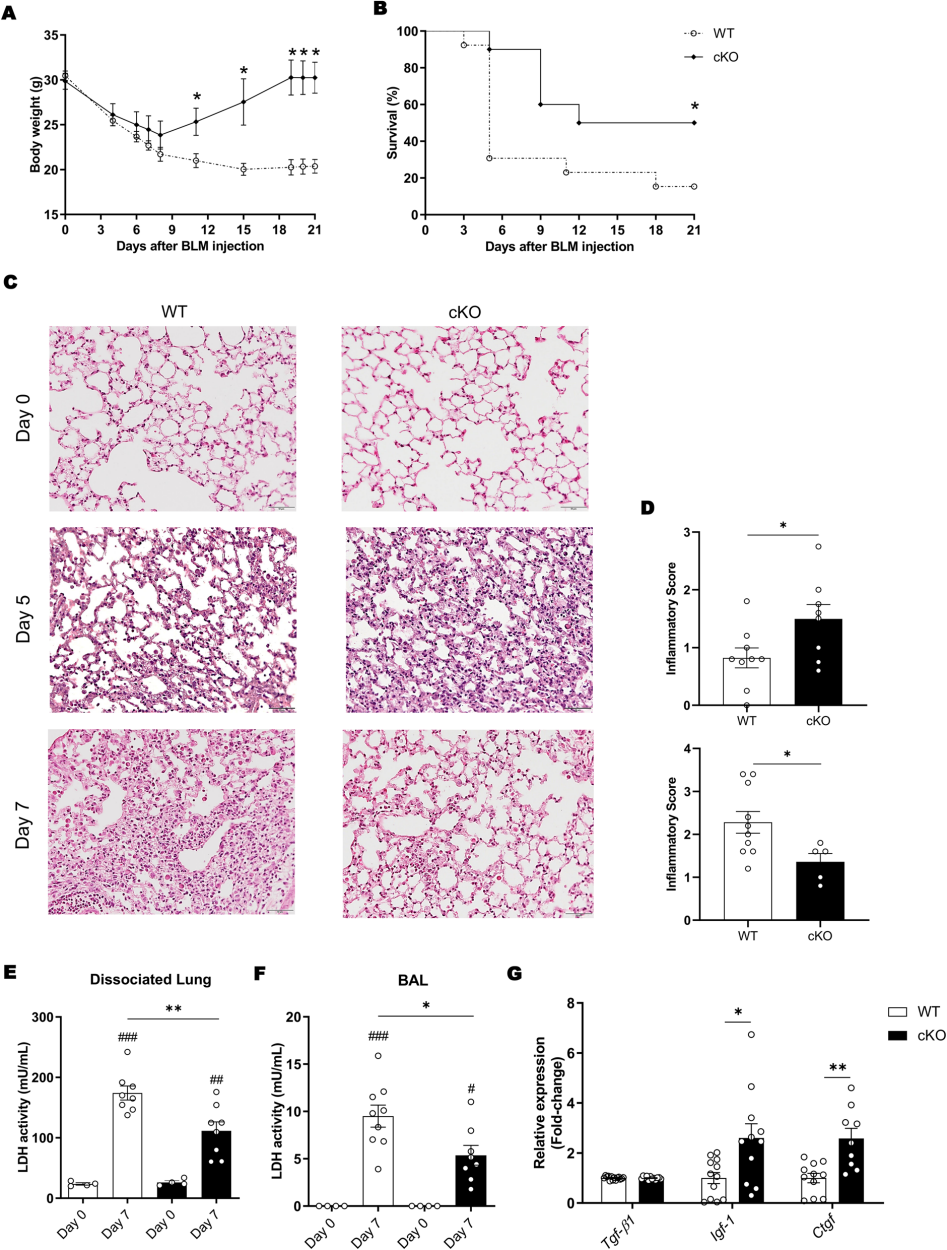
Deletion of *c-Met* from immune cells attenuates lung injury following BLM administration. WT and cKO mice administered intratracheally with BLM were followed for (**A**) weight change and (**B**) survival. *n* = 5–24 mice *per* group. (**C**) Representative images of H&E-stained lung sections from vehicle-administered mice, and on days 5 and 7 after BLM injection and (**D**) respective scoring. Original magnification × 20, scale bar 50 µm. LDH activity in the (**E**) dissociated tissue and (**F**) BAL supernatant at 7 days after BLM injection. (**G**) Gene expression of repair-associated molecules (*Tgf-β1*, *Igf-1*, *Ctgf*) in the lung at 7 days after BLM injection. *n* = 12–14 *per* group. Data are expressed as mean ± SEM and were analyzed by Student’s two-tailed t test (**A**, **D**, **G**), log-rank test (**B**) or with one-way ANOVA with Tukey’s multiple comparisons test (**E**, **F**). *p < 0.05, **p < 0.01. Similar results were obtained in three independent experiments

To investigate the impact of c-MET signaling in immune cells on the severity of BLM-induced PF, we analyzed the body weight and survival of WT mice and mice with *c-Met* deletion in immune cells, cKO, until day 21 after BLM administration (Fig. 1A). As shown in Fig. S4, when comparing WT and cKO mice at day 7, c-MET expression was effectively deleted in lung immune cells in the BLM-induced PF mouse model, rather than in the non-immune lung populations, specifically epithelial and endothelial cells, in which its expression remains unaltered. While both WT and cKO mice experienced weight lost until day 7 following BLM injection, cKO mice showed a significant recovery in body weight after day 7 (Fig. 2A). Survival analysis revealed that only 20% of WT mice survived, with the highest mortality rate on day 5, whereas 60% of cKO mice survived after BLM injection (Fig. 2B).

As the most significant differences in survival and weight recovery between animal groups occurred on day 5 and 7, we explored the alterations in BLM-induced inflammation (Fig. 1A). On day 5 after BLM administration, cKO mice exhibited slightly more inflammatory infiltration in the lungs compared to WT animals, as confirmed by a significant increase in the inflammatory score displayed by cKO mice (Fig. 2C, D). Interestingly, on day 7 after BLM administration, cKO mice presented fewer and smaller lung inflammatory infiltrates than WT mice, which had extensive lung inflammation (Fig. 2C, D). To assess tissue and cell cytotoxicity, we measured LDH activity, which was significantly reduced in dissociated lung and BAL supernatants of cKO mice 7 days after BLM injection (Fig. 2E, F). Indication of less tissue injury was found in cKO mice, so we examined the influence of c-MET signaling in immune cells on the repair environment. Notably, cKO mice presented significantly increased levels of repair-associated genes, *Igf-1* and *Ctgf,* on day 7 after BLM administration, suggesting a more pro-repair environment (Fig. 2G).

Taken together, these results demonstrate that *c-Met* deletion in immune cells leads to an earlier inflammatory cell infiltration, which results in a less cytotoxic and more proficient pro-repair environment during BLM-induced lung inflammation.

### Deletion of *c-Met* from immune cells triggers their early accumulation and restrained the proinflammatory environment

**Fig. 3:**
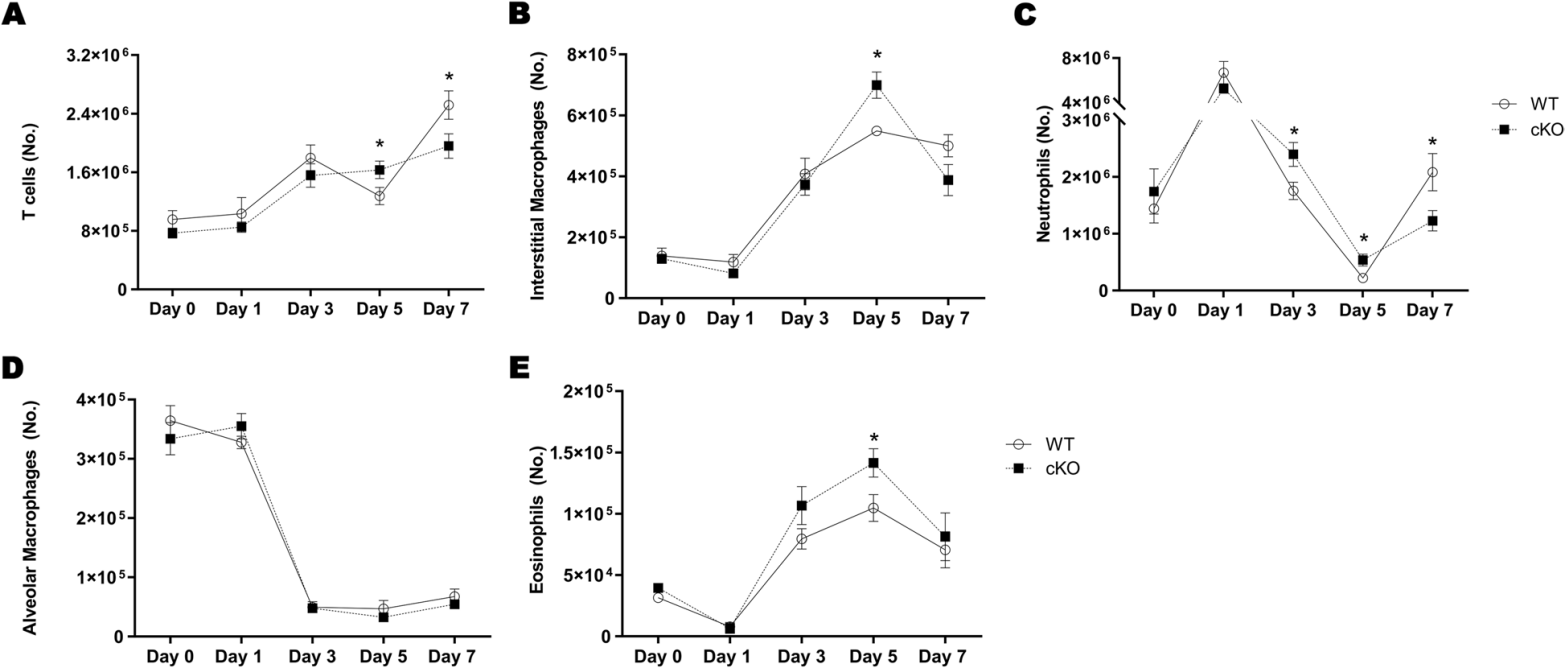
*c-Met* deletion in immune cells promotes early inflammatory cells recruitment after BLM administration. Number of (**A**) T cells, (**B**) interstitial macrophages, (**C**) neutrophils, (**D**) alveolar macrophages and (**E**) eosinophils. Cell numbers were calculated based on the total number of cells in the dissociated lung. *n* = 8–10 *per* group. Data are expressed as mean ± SEM. and were analyzed by Student’s two-tailed t test. *p < 0.05. Similar results were obtained in three independent experiments

To better characterize the kinetics of immune cell populations in the lung, we performed flow cytometry analyses during the BLM-induced inflammatory phase (Fig. 1A). The total leukocyte population was gated based on CD45 expression, then the alveolar macrophage population was readily identified based on the expression of SiglecF, CD64, CD11c and the absence of CD11b. Neutrophils exclusively express Ly-6G, allowing their identification, and eosinophils were identified based on their expression of SiglecF, absence of CD11c and after gating out alveolar macrophages, which are the only cells other than eosinophils that express SiglecF. Identification of interstitial macrophages was based on their expression of CD64, CD11b, CD11c, MHCII and absence of CD24, and T cells were identified by the expression of CD3 (Figure S1). Interestingly, in cKO mice, we observed an early accumulation of immune cell populations that would normally exhibit c-MET expression induction. The number of T cells, interstitial macrophages and eosinophils were significantly increased in the lungs of cKO mice on day 5 after injection of BLM (Fig. 3A, B, E). Of note, the number of neutrophils significantly increased in the lungs of cKO mice at 3 and 5 days after BLM injection (Fig. 3C). In contrast, and in accordance with the reduced inflammatory score observed on day 7 after BLM administration, the number of T cells and neutrophils significantly decreased in the lungs of cKO mice (Fig. 3A, C). No differences were found in the number of alveolar macrophages at any time point (Fig. 3D).

**Fig. 4:**
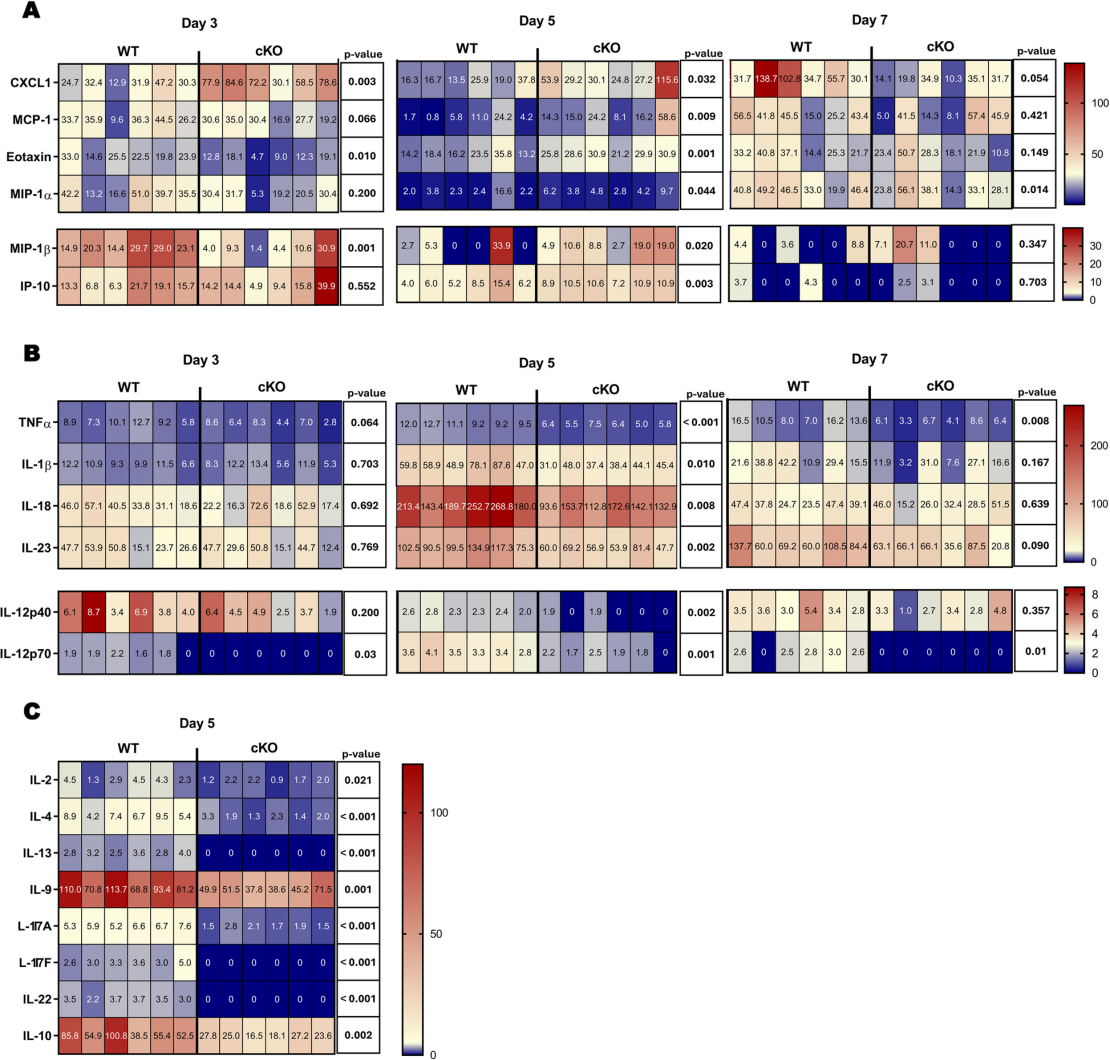
Boosted leukocyte chemoattraction and restrained proinflammatory milieu in cKO mice lung mainly on day 5 following BLM administration. Heatmap of the concentration, pg/mL, of (**A**) chemokines and (**B**) proinflammatory cytokines in lung on day 3, 5 and 7 and (**C**) interleukins associated with inflammatory cells phenotypic activation in lung on day 5 after bleomycin administration. n = 6 *per* group. *P*value for each comparation is reported, Student’s two-tailed t test

To confirm that the accumulation of immune cells was a result of alterations in chemotactic signals, we analyzed lung chemokine levels. Consistent with previous results of immune cell accumulation in the lung, the levels of chemokines for neutrophils, monocytes, macrophages, T cells and eosinophils, namely CXCL1 (KC), MCP-1 (CCL2), eotaxin (CCL11), MIP-1α (CCL3), MIP-1β (CCL4) and IFN-gamma-inducible protein of 10 kDa (IP-10/CXCL10) were significantly increased in the lungs of cKO mice on day 5 post-BLM administration, while some of these chemokines were significantly decreased on day 7 (Fig. 4A). Concordantly, the levels of thymus- and activation-regulation chemokine (TARC), a selective chemoattractant for T cells, and CXCL1 were also found to be increased in the BAL fluid of cKO mice at day 5 and decreased at day 7 (Fig. S5A and B). The early accumulation of immune cells was concomitant with the paradoxically attenuated proinflammatory environment in cKO mice. Of note, TNF-α, IL-1β, IL-18, IL-23, IL-12p40 and IL-12p70 levels were reduced in cKO mice, mostly on day 5 (Fig. 4B). Concordantly, the levels of IL-6 and IL-12p40 in the BAL fluid were also found to be reduced in cKO mice mainly at day 7 and 5, respectively (Fig. S5C and D). To clarify whether this attenuated proinflammatory environment was associated with changes in the phenotypic activation of immune cells, we assessed the levels of cytokines related to immune function, which showed a decrease in cKO mice on day 5 (Fig. 4C). The remaining cytokines measured in the multiplex assay were unaffected by *c-Met* deficiency at any time point examined (data not shown).

These data highlight the cellular immune alterations related with c-MET signaling in immune cells. Notably, *c-Met* deletion in immune cells leads to a restrained proinflammatory environment, along with a balanced phenotypic activation of inflammatory cells during BLM-induced lung inflammation.

### Lung fibrosis is attenuated by *c-Met* deletion in immune cells

**Fig. 5:**
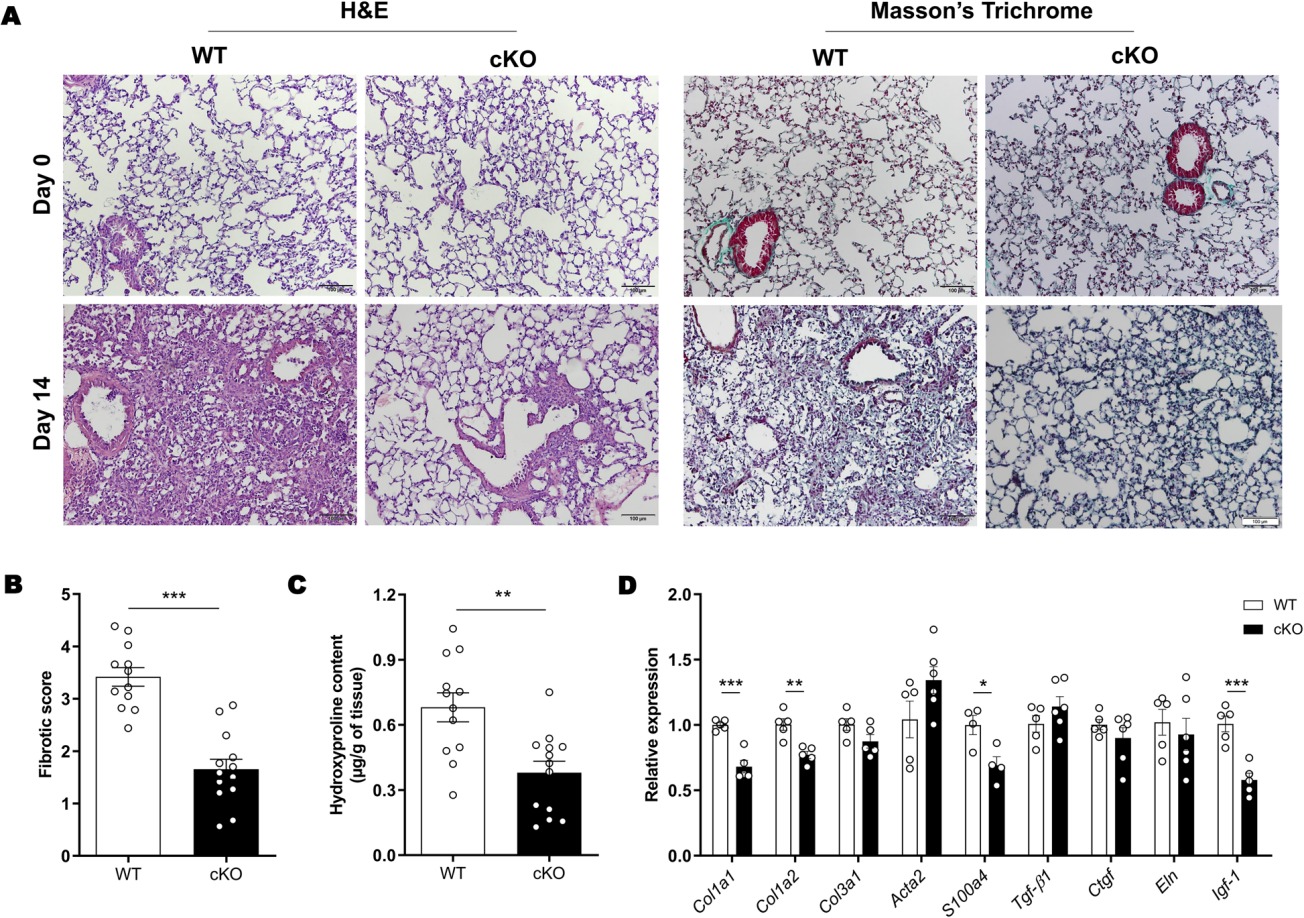
Attenuated lung fibrosis in cKO mice on day 14 following BLM administration. (**A**) Representative images of lung sections stained with H&E and Masson’s trichrome. Original magnification × 10, scale bar 100 µm. (**B) **Ashcroft scoring of lung fibrosis. (**C**) Hydroxyproline levels in lungs. (**D**) Gene expression of profibrotic mediators in whole-lung samples. *n* = 12–14 *per* group (**A**-**C**) or data representative of one of three independent experiments *n* = 6 *per* group (**D**). Data are expressed asmean ± SEM and were analyzed by Student’s two-tailed t test. *p < 0.05, **p < 0.01, ***p < 0.001. Similar results were obtained in three independent experiments

Then, we decided to clarify whether these alterations impacted the establishment of BLM-induced fibrosis (Fig. 1A). Histological analysis on day 14 after BLM administration revealed multifocal fibrotic pulmonary lesions, alveolar architecture destruction and loss of alveolar surface area in WT animals (Fig. 5A). In contrast, cKO mice displayed preserved alveolar architecture, alveolar surface area and thinner alveolar walls (Fig. 5A). To further assess fibrosis, we analyzed collagen deposition in lung sections using Masson’s trichrome staining. The pulmonary interstitium from cKO mice displayed less collagen fiber deposition compared to WT mice, indicating reduced collagen accumulation in cKO mice (Fig. 5A). In accordance with histological evaluation, the Ashcroft score was significantly lower in cKO mice (Fig. 5B). Additionally, hydroxyproline levels, a collagen component, were significantly decreased in the lungs of cKO mice at 14 days post-BLM treatment (Fig. 5C). Furthermore, cKO mice exhibited significantly lower expression levels of fibrotic markers (*Col1a1*, *Col1a2*, S*100a4* and *Igf-1)* at 14 days after BLM injection (Fig. 5D).

Taken together, these data clearly demonstrate that the deletion of *c-Met* in immune cells promotes the attenuation of PF, suggesting that c-MET expression in immune cells boosts the fibrogenesis process.

### Lung T cells from patients with fibrosing ILDs show high expression of c-MET

**Fig. 6:**
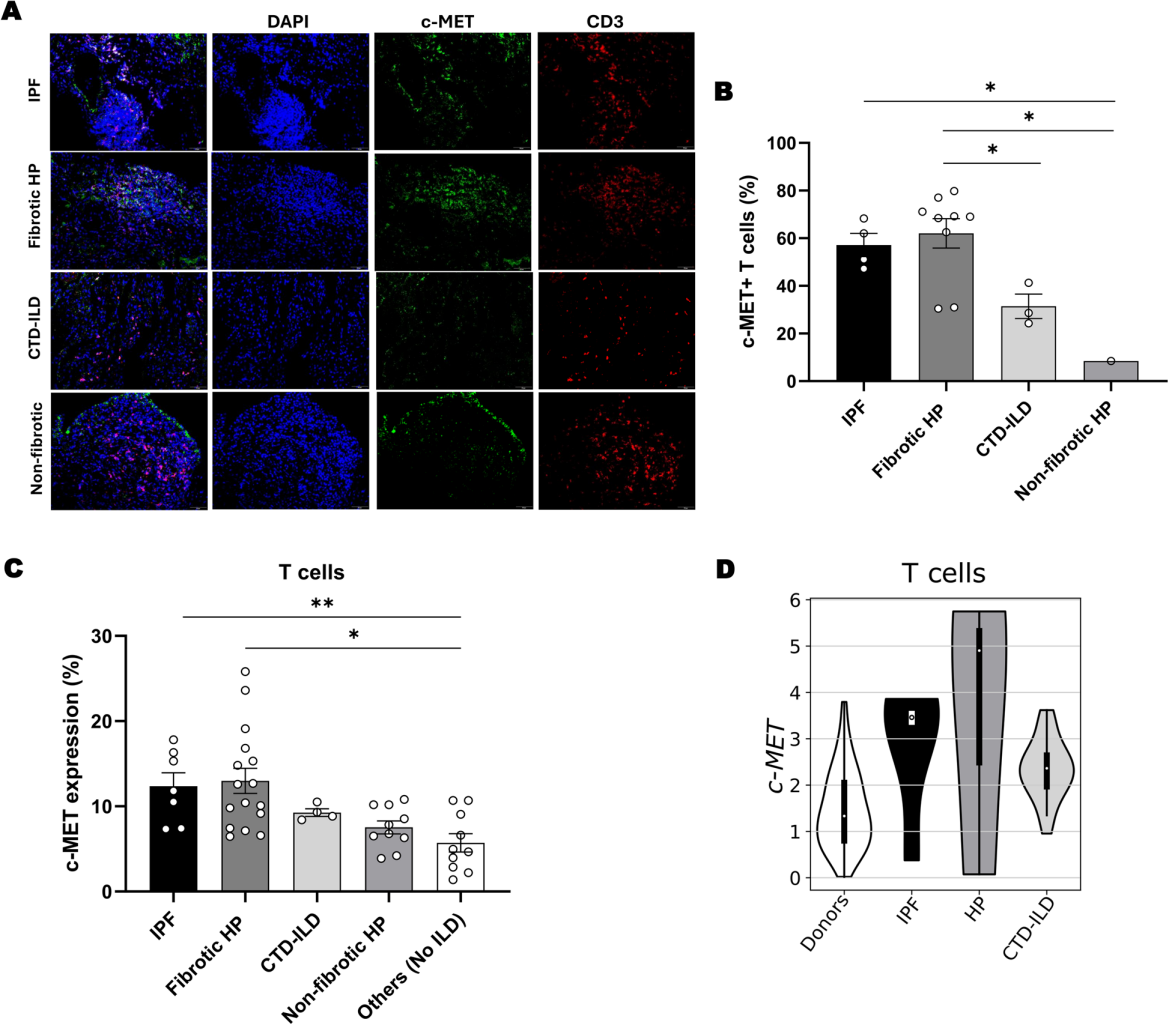
c-MET expression is augmented in T cells in fibrosis-related ILDs. (**A**) Representative images of immunofluorescence on c-MET (green) in T cells (red) and (**B**) respective expression quantification on human lung sections from a cohort of patients. Original magnification × 20, scale bar 50 µm. (**C**) Percentage of T cells with c-MET expression, quantified by flow cytometry on cryopreserved BAL samples from a cohort of patients. (**D**) Plot representing expression of *c-MET* in T cells from donor and ILD patients. *n* = 1–16 *per* group. Data are expressed as mean ± SEM and were analyzed with Kruskal–Wallis test with Dunn's multiple comparisons test. *p < 0.05, **p < 0.01

Finally, to assess the clinical relevance of c-MET signaling in ILDs, we performed immunofluorescence analysis of c-MET on lung cryobiopsies from a patient cohort of Hospital de São João (Table S2). We observed enhanced c-MET expression in T cells from patients with fibrosing conditions, mainly IPF and fibrotic HP, compared to CTD-ILD and non-fibrotic HP patients (Fig. 6A, B). Furthermore, flow cytometry analysis of cryopreserved BAL samples from an additional cohort of Hospital de São João patients (Table S3), confirmed elevated c-MET expression in T cells of fibrotic patients (Fig. 6C). Of note, a separate group of patients with lung disease other than ILDs exhibited the lowest levels of c-MET expression in T cells (Fig. 6C). Bioinformatic analysis of single-cell RNA sequencing data from lung biopsies of healthy donors and ILD patients, including IPF, HP and CTD-ILD [44] further supported our findings. Accordingly, T cells from patients with fibrosing conditions, mainly IPF and HP, presented higher *c-MET* expression levels compared to CTD-ILD patients and healthy donors (Fig. 6D).

Regarding c-MET expression in other cell populations, such as macrophages and neutrophils, no associations with fibrotic outcome were found (Fig. S6). Macrophage c-MET expression was increased in non-hypersensitivity pneumonitis conditions, including IPF and CTD-ILD (Fig. S6A, B), while neutrophil c-MET expression levels were higher in IPF patients compared to fibrotic HP and CTD-ILD, with no expression detected in non-fibrotic HP (Fig. S6C, D).

Fig. S7 illustrates the variable pattern of c-MET expression among immune cells of ILD patients. Notably, patients with IPF and fibrotic HP present the highest percentages of c-MET^+^ T cells, IPF patients present the highest percentages of c-MET^+^ neutrophils and patients with IPF and CTD-ILD present the highest percentages of c-MET^+^ macrophages.

In summary, these data reveal an association between enhanced c-MET expression in immune cells and interstitial lung diseases, particularly indicating a potential involvement of c-MET expression in T cells underlying the development of the fibrosing condition.

## Discussion

The current therapies for pulmonary fibrosis primarily slow disease progression rather than reversing it. Previous studies have highlighted the crucial role of c-MET in regulating immune cell recruitment and effector functions in various contexts, such as inflamed tumors, *Leishmania mexicana* infection, and intestinal inflammation. Our study now demonstrates that the immune HGF/c-MET signaling pathway is a novel modulator of the progression of PF. We show that both ILD patients and mice during the acute inflammatory phase of BLM-induced PF have enhanced c-MET expression in lung immune cells. Moreover, we show that the absence of *c-Met* in immune cells reduced the severity of BLM-induced PF. This finding was correlated with an anticipated infiltration of immune cells, namely T cells, IMs, neutrophils, and eosinophils in the lungs, along with a decreased proinflammatory and cytotoxic environment. Accordingly, *c-Met* deletion in immune cells led to more controlled and faster resolution of inflammation than under normal conditions, thereby ameliorating BLM-induced PF.

Previous studies showed induction of c-MET expression in immune cells upon inflammatory stimuli [36–40, 48]. Particularly, c-MET expression levels were found to be upregulated in circulating human and mouse tumor-associated neutrophils upon inflammatory stimuli [36]. In the same line, *L. mexicana* induces c-MET expression predominantly in neutrophils [39]. Additionally, in a murine model of intestinal inflammation, c-MET expression was induced in blood and colonic neutrophils [40]. Accordingly, we now show that in a well-established murine model of PF induced by BLM, characterized by an early phase of acute lung inflammation and injury [45–47], T cells, interstitial macrophages and neutrophils upregulated c-MET expression during this phase.

Furthermore, c-MET exerts an important regulation of immune cell recruitment and functions in various inflammatory contexts. Particularly, c-MET expression in neutrophils has been shown to be required for their transmigration and nitric oxide release in inflamed tumors, which are essential for tumor cell killing [36]. Also, c-MET inhibition reduced neutrophil recruitment into T cell–inflamed tumor microenvironment and draining lymph nodes in response to cytotoxic immunotherapies [37]. In CD8^+^ T lymphocytes, c-MET expression enhances their cytolytic capacities ultimately providing greater efficacy in killing melanoma cells [38]. Additionally, a mouse model with *c-Met* deleted in neutrophils better controlled the parasite load and the lesion size after *L. mexicana* infection, once *c-Met* deletion reinforced neutrophilic ROS production after infection [39]. The same mouse model, with *c-Met* deletion in neutrophils, exhibited reduced disease severity upon induction of intestinal inflammation [40]. Furthermore, HGF treatment of c-MET-expressing M1 macrophages shift their phenotype toward M2 [48]. To further dissect the role of c-MET in the immune response following BLM administration, we used, in agreement with previous studies [36], the Tie2-Cre line to specifically delete *c-Met* from bone marrow immune cells, as limited cross-activation of c-MET was detected in endothelial cells in our mouse model of PF. Interestingly, mice with absence of *c-Met* in immune cells exhibited attenuated long-term severity of BLM-induced fibrosis, as revealed by the lower weight loss and improved survival. Moreover, mice with deletion of *c-Met* in immune cells presented reduced lung inflammation and injury at the peak of the inflammatory phase of BLM-induced PF. Interestingly, this reduced lung injury was concomitant with higher expression levels of repair-associated growth factors, namely *Igf-1* and *Ctgf*. In fact, previous studies demonstrated that these growth factors could have opposite roles depending on the time course of the disease. Particularly, in the presence of inflammation or without the premature high expression levels of TGF-β, IGF-1 production led to enhanced epithelial cell proliferation and to the absence of chronically denuded alveolar basement membranes, which together result in a constant repair process [49]. Furthermore, in skeletal muscle regeneration, IGF-1 produced by monocyte/macrophage has also been described as a potent enhancer of tissue regeneration [50]. CTGF is a well-known player downstream TGF-β. However once TGF-β is not altered, an early, transient upregulation of CTGF in the wound repair process promotes normal repair and is necessary to maintain an appropriate angiogenic process within wounds [51]. These studies suggest that when expression is confined to early tissue repair, IGF-1 and CTGF serve a pro-reparative role.

Remarkably, during BLM-induced inflammation, an early increase in infiltration of inflammatory cells into the lung of cKO mice was observed, namely the cell populations which were normally found to have induced c-MET expression, T cells, interstitial macrophages, neutrophils, and eosinophils, paradoxically with less simultaneous proinflammatory environment. Overall, *c-Met* deletion associates with lower inflammation, concomitant with a less cytotoxic, less proinflammatory and more repairing environment at the peak of inflammatory phase.

Chemokines are essential players to enhance and properly control the recruitment of inflammatory cells to injury sites. Interestingly, the expression of CXCL1, a potent neutrophil chemoattractant [52, 53], was increased in cKO mice on day 3 and 5, which was concomitant with an increased proportion of neutrophils in the lung. Subsequently, on day 7, the proportion of neutrophils in cKO mice lung was drastically decreased compared to WT, as well as the CXCL1 expression. Concomitant with increased MCP-1 expression on day 5, the proportion of macrophages was increased in cKO mice at this time point. MCP-1 is a potent chemoattractant and activator of monocytes/macrophages and is involved in macrophage activation leading to the release of inflammatory mediators and tissue damage [54, 55]. Eotaxin, known to be a potent chemoattractant for eosinophils [56], showed increased levels in cKO mice on day 5. These come in accordance with the flow cytometry results, since eosinophils proportion was higher in cKO mice at this time point. MIP-1α (CCL3), MIP-1β (CCL4) and IP-10 (CXCL10) are known chemoattractants for monocytes/macrophages and T cells [57]. Expression levels of these chemokines were augmented in cKO mice on day 5. Accordingly, both interstitial macrophages and T lymphocytes were increased in cKO mice at this time point.

The balanced production of proinflammatory cytokines and interleukins associated with the phenotypic activation of inflammatory cells is crucial for controlling inflammation at tissue injury sites. This control prevents exacerbations of damage and favor tissue repair. As so, we evaluated whether the production of cytokines and interleukins associated with inflammatory activation profile was influenced by the c-MET signaling in immune cells. Notably, the analysis of lung proinflammatory cytokines revealed diminished levels in cKO mice compared to WT, mainly on day 5. Furthermore, levels of interleukins produced by inflammatory cells upon activation were lower in the lung of cKO mice. This result suggests that the reduced lung injury in cKO mice is a consequence of more controlled inflammatory recruitment and activation, and diminished proinflammatory environment, which results in lung repair rather than fibrosis progression. In fact, our data at the peak of the fibrotic phase of BLM-induced PF, that occurs on day 14, demonstrated a decrease in fibrotic lesion extension, collagen deposition and in the levels of a profibrotic factor, Igf-1 [58], associated with *c-Met* deletion in the immune cells.

Altogether, our findings reveal that c-MET signaling in immune cells is enhancing their uncontrolled recruitment and activation toward a more proinflammatory and profibrotic phenotype, exacerbating lung injury and ultimately culminating in fibrosis in the BLM-induced PF mouse model.

**Fig. 7:**
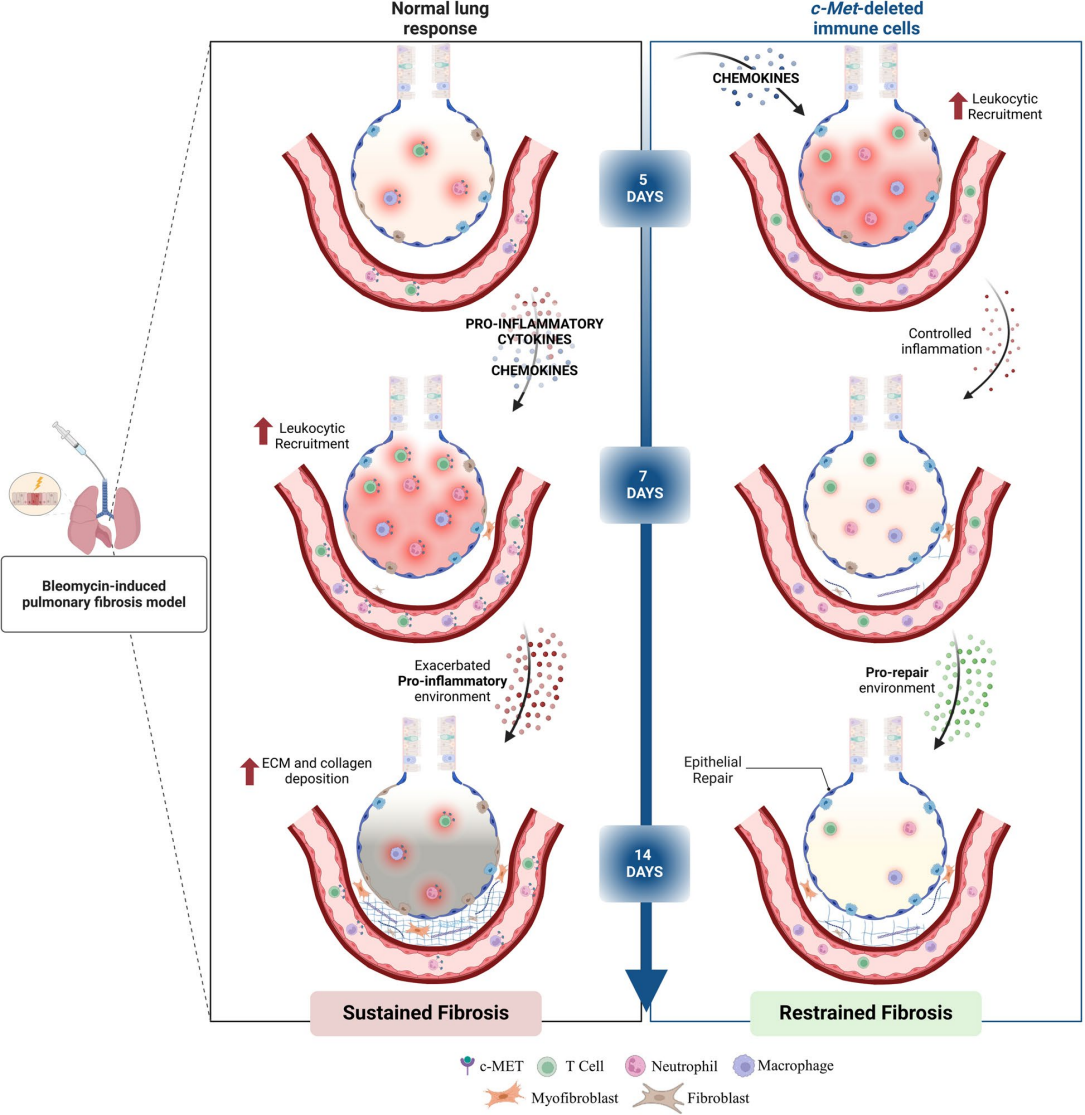
Schematic illustration of the importance of c-MET in immune cells during BLM-induced PF progression. Lung injury is induced by BLM leading to uncontrolled inflammatory recruitment, followed by excessive deposition of extracellular matrix proteins, as collagen. Absence of *c-Met* in immune cells resulted in anticipated inflammatory recruitment, attenuated proinflammatory environment, leading to higher epithelial repair, which later culminates in lower collagen deposition and prevents fibrosis

As illustrated in Fig. 7, our results show that *c-Met* deletion in immune cells significantly ameliorated BLM-induced lung fibrosis. The attenuation of lung fibrosis can be attributed to the anticipated inflammatory recruitment and concomitant attenuated proinflammatory environment, ultimately leading to enhanced epithelial repair. Overall, suggesting that c-MET signaling in immune cells deregulates inflammatory recruitment and activation, ultimately leading to pulmonary fibrosis.

ILDs include a heterogenous group of parenchyma disorders associated with variable lung inflammatory patterns and fibrosis, being IPF the prototypical fibroproliferative disorder [7]. Additionally, other common ILDs can also develop a fibrotic condition, namely HP and CTD-ILD [3, 59–62]. Although patients with CTD-ILD can be also categorized as non-fibrotic or fibrotic, in our study this distinction was not made. Immune cells have been involved in the pathogenesis of ILDs [63–65], with various populations contributing to the progression of ILD, particularly macrophages [44, 66–68] and lymphoid cells [64, 69, 70]. Our results show that T cells, macrophages and neutrophils present enhanced c-MET expression in specific ILDs. Specifically, c-MET expression is higher in T cells from patients with fibrotic conditions, mainly IPF and fibrotic HP. In contrast, macrophages display increased c-MET expression in non-HP conditions, namely IPF and CTD-ILD. Furthermore, neutrophils exhibited elevated c-MET expression in IPF patients compared to those with other fibrotic conditions. Concordantly, a bioinformatics analysis of single-cell RNA sequencing of biopsies from healthy donors and patients with ILDs, revealed an enrichment of c-MET expressing T cells in patients with fibrosing conditions, mainly IPF and HP. Thus, suggesting a potential involvement of c-MET expression in T cells in the development of the fibrosing condition. Interestingly, in the BAL of patients with IPF, the concentration of HGF was significantly higher than in normal controls [71–74]. Since c-MET signaling is classically activated by the binding of its primary ligand, HGF, the receptor and the respective signaling pathway will therefore be overactivated under fibrotic conditions. In fact, this is corroborated by a recent study that confirms an upregulation of c-MET in the fibrotic foci either in IPF and fibrotic hypersensitivity pneumonia, reinforcing its importance in the pathogenesis of pulmonary fibrosis [75]. As such, the expression of c-MET in lung immune cells might be induced by HGF under fibrotic conditions, reinforcing their proinflammatory and cytotoxic capacities which contributes to the establishment of tissue fibrosis.

Together, both mice and human data highlight the contribution of c-MET expressing immune cells to the progression of fibrosing condition. Therefore, selective blockage of c-MET pathway in immune cells emerges as an attractive therapeutic strategy to control tissue damage triggered by inflammation.

## Conclusions

Our results demonstrate that c-MET signaling pathway modulates uncontrolled recruitment of immune cells and activation toward a proinflammatory and profibrotic phenotype, exacerbating lung injury and ultimately culminating in lung fibrosis. Taken together our findings suggest the potential involvement of c-MET in the development of fibrosing disease. Thus, highlighting that c-MET signaling pathway may be a promising target for therapeutic strategies aimed at the treatment of lung fibrosis.

### Supplementary Information

Supplementary Material 1.

Supplementary Material 2.

## Data Availability

No datasets were generated or analysed during the current study.

## Abbreviations

BAL: Bronchoalveolar lavage
BLM: Bleomycin
CTD-ILD: Connective tissue disease-associated interstitial lung disease
HGF: Hepatocyte growth factor
HP: Hypersensitivity pneumonitis
ILD: Interstitial lung disease
IMs: Interstitial macrophages
LDH: Lactate dehydrogenase
MFI: Mean fluorescent intensity
IPF: Idiopathic pulmonary fibrosis
PF: Pulmonary fibrosis

## References

[1] McLean-Tooke A, Moore I, Lake F (2019). Idiopathic and immune-related pulmonary fibrosis: diagnostic and therapeutic challenges. Clin Transl Immunol.

[2] Xu Y, Dong J, An W, Lv X, Yin X, Zhang J-Z (2020). Clinical and computed tomographic imaging features of novel coronavirus pneumonia caused by SARS-CoV-2. J Infect.

[3] Salisbury ML, Myers JL, Belloli EA, Kazerooni EA, Martinez FJ, Flaherty KR (2017). Diagnosis and treatment of fibrotic hypersensitivity pneumonia. Where we stand and where we need to go. Am J Respir Crit Care Med.

[4] Wells AU, Denton CP (2014). Interstitial lung disease in connective tissue disease—mechanisms and management. Nat Rev Rheumatol.

[5] Cottin V (2016). Idiopathic interstitial pneumonias with connective tissue diseases features: a review. Respirology.

[6] Mira-Avendano I, Abril A, Burger CD, Dellaripa PF, Fischer A, Gotway MB (2019). Interstitial lung disease and other pulmonary manifestations in connective tissue diseases. Mayo Clin Proc.

[7] Ley B, Collard HR, King TE (2011). Clinical course and prediction of survival in idiopathic pulmonary fibrosis. Am J Respir Crit Care Med.

[8] Raghu G (2013). Treatment of idiopathic pulmonary fibrosis with Ambrisentan. Ann Intern Med.

[9] Shulgina L, Cahn AP, Chilvers ER, Parfrey H, Clark AB, Wilson ECF (2013). Treating idiopathic pulmonary fibrosis with the addition of co-trimoxazole: a randomised controlled trial. Thorax.

[10] Raghu G, Collard HR, Egan JJ, Martinez FJ, Behr J, Brown KK (2011). An official ATS/ERS/JRS/ALAT statement: idiopathic pulmonary fibrosis: evidence-based guidelines for diagnosis and management. Am J Respir Crit Care Med.

[11] Raghu G, Rochwerg B, Zhang Y, Garcia CAC, Azuma A, Behr J (2015). An official ATS/ERS/JRS/ALAT clinical practice guideline: treatment of idiopathic pulmonary fibrosis. An update of the 2011 clinical practice guideline. Am J Respir Crit Care Med.

[12] Knudsen L, Ruppert C, Ochs M (2017). Tissue remodelling in pulmonary fibrosis. Cell Tissue Res.

[13] Fernandez IE, Eickelberg O (2012). New cellular and molecular mechanisms of lung injury and fibrosis in idiopathic pulmonary fibrosis. Lancet.

[14] Wynn TA (2011). Integrating mechanisms of pulmonary fibrosis. J Exp Med.

[15] Ricard-Blum S, Baffet G, Théret N (2018). Molecular and tissue alterations of collagens in fibrosis. Matrix Biol.

[16] Furuie H, Yamasaki H, Suga M, Ando M (1997). Altered accessory cell function of alveolar macrophages: a possible mechanism for induction of Th2 secretory profile in idiopathic pulmonary fibrosis. Eur Respir J.

[17] Hancock A, Armstrong L, Gama R, Millar A (1998). Production of interleukin 13 by alveolar macrophages from normal and fibrotic lung. Am J Respir Cell Mol Biol.

[18] Jakubzick C, Kunkel SL, Puri RK, Hogaboam CM (2004). Therapeutic targeting of IL-4- and IL-13-responsive cells in pulmonary fibrosis. Immunol Res.

[19] Wynn TA (2015). Type 2 cytokines: mechanisms and therapeutic strategies. Nat Rev Immunol.

[20] Chung SI, Horton JA, Ramalingam TR, White AO, Chung EJ, Hudak KE (2016). IL-13 is a therapeutic target in radiation lung injury. Sci Rep.

[21] Puttur F, Gregory LG, Lloyd CM (2019). Airway macrophages as the guardians of tissue repair in the lung. Immunol Cell Biol.

[22] Hesketh M, Sahin KB, West ZE, Murray RZ (2017). Macrophage phenotypes regulate scar formation and chronic wound healing. Int J Mol Sci.

[23] Byrne AJ, Mathie SA, Gregory LG, Lloyd CM (2015). Pulmonary macrophages: key players in the innate defence of the airways. Thorax.

[24] Joshi N, Walter JM, Misharin AV (2018). Alveolar macrophages. Cell Immunol.

[25] Gibbings SL, Thomas SM, Atif SM, McCubbrey AL, Desch AN, Danhorn T (2017). Three unique interstitial macrophages in the murine lung at steady state. Am J Respir Cell Mol Biol.

[26] Song E, Ouyang N, Hörbelt M, Antus B, Wang M, Exton MS (2000). Influence of alternatively and classically activated macrophages on fibrogenic activities of human fibroblasts. Cell Immunol.

[27] Libório-Ramos S, Barbosa-Matos C, Fernandes R, Borges-Pereira C, Costa S (2023). Interstitial macrophages lead early stages of bleomycin-induced lung fibrosis and induce fibroblasts activation. Cells.

[28] Murray PJ (2017). Macrophage polarization. Annu Rev Physiol.

[29] Zhang L, Wang Y, Wu G, Xiong W, Gu W, Wang C-Y (2018). Macrophages: friend or foe in idiopathic pulmonary fibrosis?. Respir Res.

[30] Kimura T, Nada S, Takegahara N, Okuno T, Nojima S, Kang S (2016). Polarization of M2 macrophages requires Lamtor1 that integrates cytokine and amino-acid signals. Nat Commun.

[31] Byrne AJ, Maher TM, Lloyd CM (2016). Pulmonary macrophages: a new therapeutic pathway in fibrosing lung disease?. Trends Mol Med.

[32] Kolaczkowska E, Kubes P (2013). Neutrophil recruitment and function in health and inflammation. Nat Rev Immunol.

[33] Gregory AD, Kliment CR, Metz HE, Kim K-H, Kargl J, Agostini BA (2015). Neutrophil elastase promotes myofibroblast differentiation in lung fibrosis. J Leukoc Biol.

[34] Takemasa A, Ishii Y, Fukuda T (2012). A neutrophil elastase inhibitor prevents bleomycin-induced pulmonary fibrosis in mice. Eur Respir J.

[35] Kinder BW, Brown KK, Schwarz MI, Ix JH, Kervitsky A, King TE (2008). Baseline BAL neutrophilia predicts early mortality in idiopathic pulmonary fibrosis. Chest.

[36] Finisguerra V, Di Conza G, Di Matteo M, Serneels J, Costa S, Thompson AAR (2015). MET is required for the recruitment of anti-tumoural neutrophils. Nature.

[37] Glodde N, Bald T, van den Boorn-Konijnenberg D, Nakamura K, O’Donnell JS, Szczepanski S (2017). Reactive neutrophil responses dependent on the receptor tyrosine kinase c-MET limit cancer immunotherapy. Immunity.

[38] Benkhoucha M, Molnarfi N, Kaya G, Belnoue E, Bjarnadóttir K, Dietrich P (2017). Identification of a novel population of highly cytotoxic c-Met-expressing CD8 + T lymphocytes. EMBO Rep.

[39] Passelli K, Prat-Luri B, Merlot M, Goris M, Mazzone M, Tacchini-Cottier F (2022). The c-MET receptor tyrosine kinase contributes to neutrophil-driven pathology in cutaneous leishmaniasis. PLoS Pathog.

[40] Stakenborg M, Verstockt B, Meroni E, Goverse G, De Simone V, Verstockt S (2020). Neutrophilic HGF-MET signalling exacerbates intestinal inflammation. J Crohn’s Colitis.

[41] Gori S, Alcain J, Vanzulli S, Moreno Ayala MA, Candolfi M, Jancic C (2019). Acetylcholine-treated murine dendritic cells promote inflammatory lung injury. PLoS One.

[42] Ashcroft T, Simpson JM, Timbrell V (1988). Simple method of estimating severity of pulmonary fibrosis on a numerical scale. J Clin Pathol.

[43] Livak KJ, Schmittgen TD (2001). Analysis of relative gene expression data using real-time quantitative PCR and the 2−ΔΔCT method. Methods.

[44] Reyfman PA, Walter JM, Joshi N, Anekalla KR, McQuattie-Pimentel AC, Chiu S (2019). Single-cell transcriptomic analysis of human lung provides insights into the pathobiology of pulmonary fibrosis. Am J Respir Crit Care Med.

[45] Moeller A, Ask K, Warburton D, Gauldie J, Kolb M (2008). The bleomycin animal model: a useful tool to investigate treatment options for idiopathic pulmonary fibrosis?. Int J Biochem Cell Biol.

[46] Mouratis MA, Aidinis V (2011). Modeling pulmonary fibrosis with bleomycin. Curr Opin Pulm Med.

[47] Moore BB, Hogaboam CM (2008). Murine models of pulmonary fibrosis. Am J Physiol Cell Mol Physiol.

[48] Nishikoba N, Kumagai K, Kanmura S, Nakamura Y, Ono M, Eguchi H (2020). HGF-MET signaling shifts M1 macrophages toward an M2-like phenotype through PI3K-mediated induction of arginase-1 expression. Front Immunol.

[49] Krein PM, Winston BW (2002). Roles for insulin-like growth factor I and transforming growth factor-β in fibrotic lung disease. Chest.

[50] Tonkin J, Temmerman L, Sampson RD, Gallego-Colon E, Barberi L, Bilbao D (2015). Monocyte/macrophage-derived IGF-1 orchestrates murine skeletal muscle regeneration and modulates autocrine polarization. Mol Ther.

[51] Alfaro MP, Deskins DL, Wallus M, DasGupta J, Davidson JM, Nanney LB (2013). A physiological role for connective tissue growth factor in early wound healing. Lab Investig.

[52] Russo RC, Guabiraba R, Garcia CC, Barcelos LS, Roffê E, Souza ALS (2009). Role of the chemokine receptor CXCR2 in bleomycin-induced pulmonary inflammation and fibrosis. Am J Respir Cell Mol Biol.

[53] Cheng I, Liu C, Lin J, Hsu T-W, Hsu J, Li AF-Y (2019). Particulate matter increases the severity of bleomycin-induced pulmonary fibrosis through KC-mediated neutrophil chemotaxis. Int J Mol Sci.

[54] Baran CP, Opalek JM, McMaken S, Newland CA, O’Brien JM, Hunter MG (2007). Important roles for macrophage colony-stimulating factor, CC chemokine ligand 2, and mononuclear phagocytes in the pathogenesis of pulmonary fibrosis. Am J Respir Crit Care Med.

[55] Okuma T, Terasaki Y, Kaikita K, Kobayashi H, Kuziel WA, Kawasuji M (2004). C-C chemokine receptor 2 (CCR2) deficiency improves bleomycin-induced pulmonary fibrosis by attenuation of both macrophage infiltration and production of macrophage-derived matrix metalloproteinases. J Pathol.

[56] Huaux F, Gharaee-Kermani M, Liu T, Morel V, McGarry B, Ullenbruch M (2005). Role of Eotaxin-1 (CCL11) and CC chemokine receptor 3 (CCR3) in bleomycin-induced lung injury and fibrosis. Am J Pathol.

[57] Craig VJ, Quintero PA, Fyfe SE, Patel AS, Knolle MD, Kobzik L (2013). Profibrotic activities for matrix metalloproteinase-8 during bleomycin-mediated lung injury. J Immunol.

[58] Hung CF, Rohani MG, Lee S, Chen P, Schnapp LM (2013). Role of IGF-1 pathway in lung fibroblast activation. Respir Res.

[59] Hamblin M, Prosch H, Vašáková M (2022). Diagnosis, course and management of hypersensitivity pneumonitis. Eur Respir Rev.

[60] Selman M, Pardo A, King TE (2012). Hypersensitivity pneumonitis. Am J Respir Crit Care Med.

[61] Raghu G, Remy-Jardin M, Ryerson CJ, Myers JL, Kreuter M, Vasakova M (2020). Diagnosis of hypersensitivity pneumonitis in adults: an official ATS/JRS/ALAT clinical practice guideline. Am J Respir Crit Care Med.

[62] Fernández Pérez ER, Travis WD, Lynch DA, Brown KK, Johannson KA, Selman M (2021). Executive summary. Chest.

[63] Yanagihara T, Sato S, Upagupta C, Kolb M (2019). What have we learned from basic science studies on idiopathic pulmonary fibrosis?. Eur Respir Rev.

[64] Serezani APM, Pascoalino BD, Bazzano JMR, Vowell KN, Tanjore H, Taylor CJ (2022). Multiplatform single-cell analysis identifies immune cell types enhanced in pulmonary fibrosis. Am J Respir Cell Mol Biol.

[65] Yanagihara T, Inoue Y (2020). Insights into pathogenesis and clinical implications in myositis-associated interstitial lung diseases. Curr Opin Pulm Med.

[66] Wynn TA, Vannella KM (2016). Macrophages in tissue repair, regeneration, and fibrosis. Immunity.

[67] Morse C, Tabib T, Sembrat J, Buschur KL, Bittar HT, Valenzi E (2019). Proliferating SPP1/MERTK-expressing macrophages in idiopathic pulmonary fibrosis. Eur Respir J.

[68] Adams TS, Schupp JC, Poli S, Ayaub EA, Neumark N, Ahangari F (2020). Single-cell RNA-seq reveals ectopic and aberrant lung-resident cell populations in idiopathic pulmonary fibrosis. Sci Adv.

[69] Celada LJ, Kropski JA, Herazo-Maya JD, Luo W, Creecy A, Abad AT (2018). PD-1 up-regulation on CD4 + T cells promotes pulmonary fibrosis through STAT3-mediated IL-17A and TGF-β1 production. Sci Transl Med.

[70] d’Alessandro M, Bergantini L, Cameli P, Fanetti M, Alderighi L, Armati M (2021). Immunologic responses to antifibrotic treatment in IPF patients. Int Immunopharmacol.

[71] Sakai T, Satoh K, Matsushima K, Shindo S, Abe S, Abe T (1997). Hepatocyte growth factor in bronchoalveolar lavage fluids and cells in patients with inflammatory chest diseases of the lower respiratory tract: detection by RIA and in situ hybridization. Am J Respir Cell Mol Biol.

[72] Przybylski G, Chorostowska-Wynimko J, Dyczek A, Wedrowska E, Jankowski M, Szpechciński A (2015). Studies of hepatocyte growth factor in bronchoalveolar lavage fluid in chronic interstitial lung diseases. Pol Arch Med Wewn.

[73] Hojo S, Fujita J, Yoshinouchi T, Yamanouchi H, Kamei T, Yamadori I (1997). Hepatocyte growth factor and neutrophil elastase in idiopathic pulmonary fibrosis. Respir Med.

[74] Yamanouchi H, Fujita J, Yoshinouchi T, Hojo S, Kamei T, Yamadori I (1998). Measurement of hepatocyte growth factor in serum and bronchoalveolar lavage fluid in patients with pulmonary fibrosis. Respir Med.

[75] Melocchi L, Cervi G, Sartori G, Gandolfi L, Jocollé G, Cavazza A (2023). Up-regulation by overexpression of c-MET in fibroblastic foci of usual interstitial pneumonia. Pathologica.

