# Supplementary material for: Deregulated immune cell recruitment orchestrated by c-MET impairs pulmonary inflammation and fibrosis
Source: Respir Res. 2024 Jun 22;25:257. doi: 10.1186/s12931-024-02884-1 (PMC11193258; doi:10.1186/s12931-024-02884-1)
Supplement: Supplementary file 1 — Supplementary Material 1. [file 12931_2024_2884_MOESM1_ESM.docx]

**Supplementary Tables**

**Table S1:** Demographics of patients who performed lung biopsy.

| **Disease**    **Characteristic** | **IPF** | **Fibrotic HP** | **CTD-ILD** | **Non-fibrotic HP** |
| --- | --- | --- | --- | --- |
| **Age, mean ± SD, years** | 60.8 ± 11.7 | 69.1 ± 6.3 | 77.0 ± 6.1 | 63.0 ± 0.0 |
| **Sex, male/female, number** | 6/0 | 2/7 | 2/1 | 0/1 |
| **Smoking status, active/never, number** | 5/1 | 3/6 | 1/2 | 0/1 |

Definition of abbreviations: IPF = idiopathic pulmonary fibrosis; HP = hypersensitivity pneumonitis; CTD‑ILD = Connective tissue disease-associated interstitial lung disease.

**Table S2:** Demographics of patients who performed bronchoalveolar lavage collection.

| **Disease**    **Characteristic** | **IPF** | **Fibrotic HP** | **CTD-ILD** | **Non-fibrotic HP** | **Others**  **(No ILD)** |
| --- | --- | --- | --- | --- | --- |
| **Age, mean ± SD, years** | 76.8 ± 6.0 | 71.3 ± 7.8 | 59.4 ± 13.0 | 61.9 ± 11.1 | 55.8 ± 11.6 |
| **Sex, male/female, number** | 6/3 | 7/11 | 2/3 | 3/8 | 5/5 |
| **Smoking status, active/former/never, number** | 0/8/1 | 1/4/13 | 1/1/3 | 0/10/1 | 3/2/5 |

Definition of abbreviations: IPF = idiopathic pulmonary fibrosis; HP = hypersensitivity pneumonitis; CTD‑ILD = Connective tissue disease-associated interstitial lung disease.

**Table S3:** Antibodies used for flow cytometry staining of mouse samples.

| Antibody | Clone | Isotype | Dilution | Conjugate | Catalog #, Company |
| --- | --- | --- | --- | --- | --- |
| Myeloid Panel | | | | | |
| CD11b | M1/70 | Rat IgG2b, κ | 1:100 | PE-Cy7 | 101216, Biolegend |
| CD11c | N418 | Armenian Hamster IgG | 1:100 | PE | 117308, Biolegend |
| CD24 | M1/69 | Rat IgG2b, κ | 1:100 | APC-Cy7 | 47-0242-82, Invitrogen |
| CD45 | 30-F11 | Rat IgG2b, κ | 1:200 | BV510 | 103138, Biolegend |
| CD64 | X54-5/7.1 | Mouse IgG1, κ | 1:100 | BV711 | 139311, Biolegend |
| F4/80 | BM8 | Rat IgG2a, κ | 1:100 | APC | 17-4801-82, Invitrogen |
| Ly6C | HK1.4 | Rat IgG2c, κ | 1:100 | PerCP-Cy5.5 | 128012, Biolegend |
| Ly6G | 1A8 | Rat IgG2a, κ | 1:100 | BV605 | 127639, Biolegend |
| MHCII | M5/114.15.2 | Rat IgG2b, κ | 1:100 | BV785 | 107645, Biolegend |
| SiglecF | 1RNM44N | Rat IgG2a, κ | 1:100 | Super Bright 645 | 64-1702-82, Invitrogen |
| c-MET | eBioclone 7 | Rat IgG1, κ | 1:50 | FITC | 11-8854-82, Invitrogen |
| Lymphoid Panel | | | | | |
| CD3 | 17A2 | Rat IgG2b, κ | 1:100 | APC | 100236, Biolegend |
| CD19 | 6D5 | Rat IgG2a, κ | 1:100 | APC-Cy7 | 115530, Biolegend |
| CD45 | 30-F11 | Rat IgG2b, κ | 1:200 | PE-Cy7 | 103114, Biolegend |
| CD326 | G8.8 | Rat IgG2a, κ | 1:100 | BV510 | 118231,  Biolegend |
| CD31 | MEC 13.3 | Rat IgG2a, κ | 1:200 | PE | 553373,  BD |
| c-MET | eBioclone 7 | Rat IgG1, κ | 1:50 | FITC | 11-8854-82, Invitrogen |
| Viability Dye | | | | | |
| Fixable  eFluor 450 |  |  | 1:1000 | Pacific Blue | 65‑0863‑14,  Ebioscience |

**Table S4:** Antibodies used for flow cytometry staining of human samples.

| Antibody | Clone | Isotype | Dilution | Conjugate | Catalog #, Company |
| --- | --- | --- | --- | --- | --- |
| Lymphoid Panel | | | | | |
| CD45 | HI30 | Mouse IgG1, κ | 1:400 | FITC | 304006, Biolegend |
| CD56 | HCD56 | Mouse IgG1, κ | 1:200 | PE | 318305, Biolegend |
| CD19 | HIB19 | Mouse IgG1, κ | 1:200 | APC-Cy7 | 302217, Biolegend |
| CD3 | OKT3 | Mouse IgG2a, κ | 1:200 | PE-Cy7 | 317333, Biolegend |
| c-MET | 3D6 | Mouse IgG1, κ | 1:50 | APC | 566014, BD |
