# Supplementary material for: Deregulated immune cell recruitment orchestrated by c-MET impairs pulmonary inflammation and fibrosis
Source: Respir Res. 2024 Jun 22;25:257. doi: 10.1186/s12931-024-02884-1 (PMC11193258; doi:10.1186/s12931-024-02884-1)
Supplement: Supplementary file 2 — Supplementary Material 2. [file 12931_2024_2884_MOESM2_ESM.docx]

**Supplementary Figures**


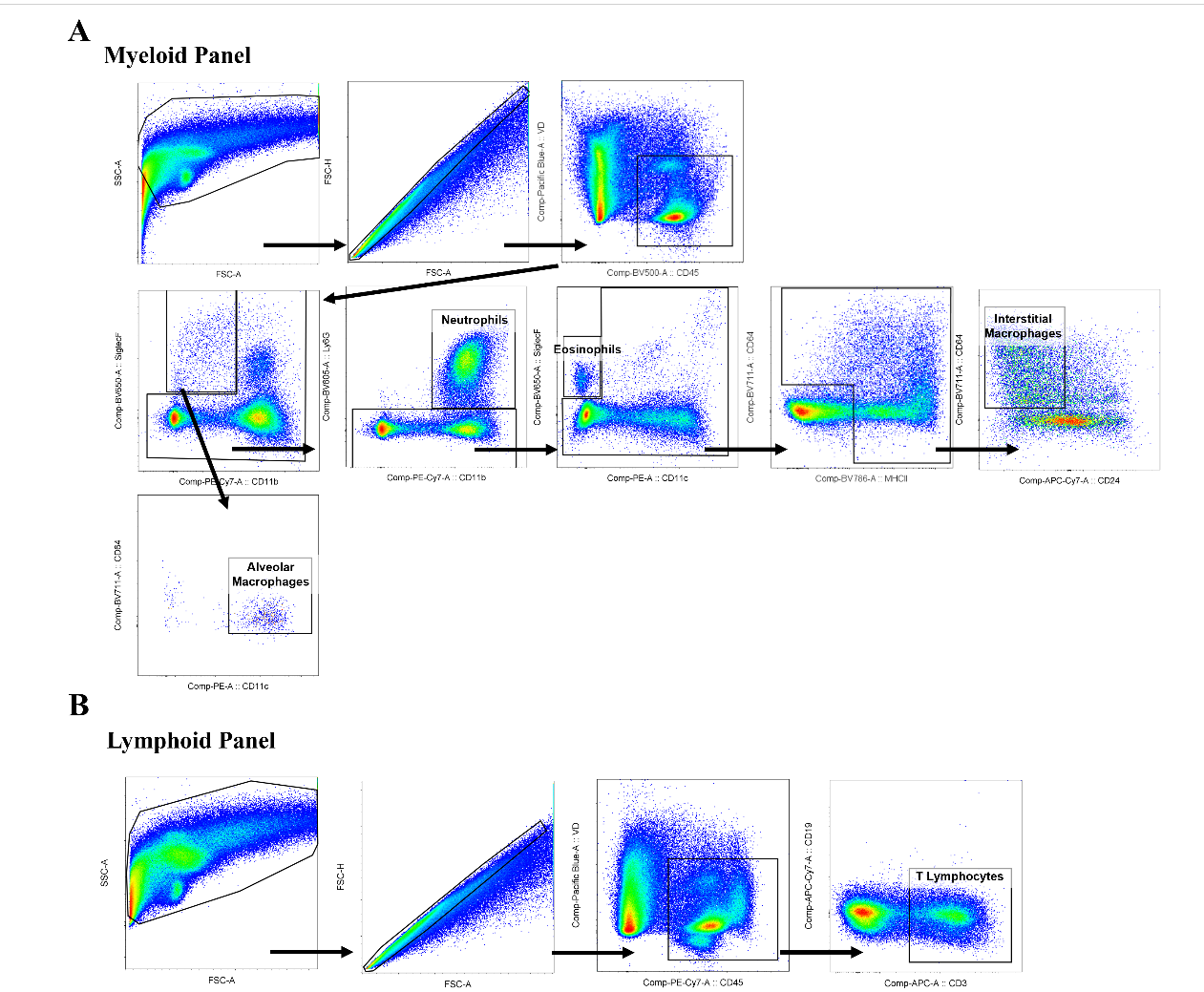


**Figure S1.** Gating strategy for the identification of myeloid and lymphoid cell populations in dissociated mice lung by flow cytometry. Initial exclusion of debris and doublets was performed using forward vs side scatter area (FSC-A vs SSC-A) and FSC-A vs FSC height (FSC-H) plot, respectively. Then live immune cells were identified by CD45 staining and negative for viable dye (VD). (A) A sequential gating strategy was used to identify myeloid populations expressing specific markers: alveolar macrophages (CD11b⁻SiglecF⁺CD11c⁺CD64^+^), neutrophils (CD11b⁺Ly-6G⁺), eosinophils (CD11c⁻SiglecF⁺), interstitial macrophages (CD11b⁺MHCII⁺CD11c⁺CD64⁺CD24⁻). (B) CD3 status was assessed to identify T cells (CD3⁺CD19ˉ). Afterward, c-MET expression was analyzed within each of the cell population mentioned.

**
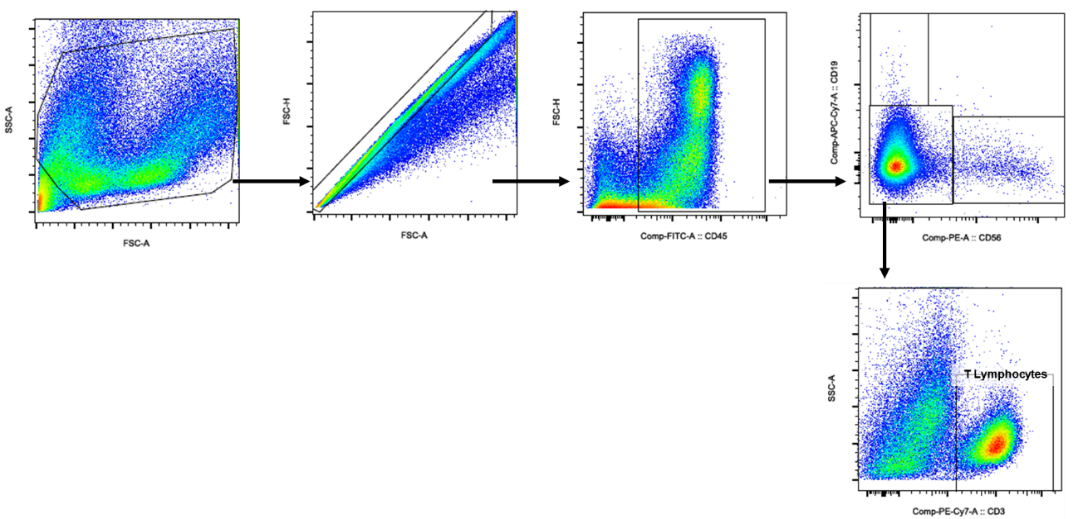
**

**Figure S2.** Gating strategy for the identification of T lymphocytes in dissociated human lung by flow cytometry. Initial exclusion of debris and doublets was performed FSC-A vs SSC‑A and FSC-A vs FSC-H plot, respectively. Then immune cells were identified by CD45 staining. CD3 status was assessed to identify T cells in double negative cells for CD19 and CD56 (CD3⁺CD19ˉ CD56ˉ). Then, c-MET expression was analyzed within this cell population.
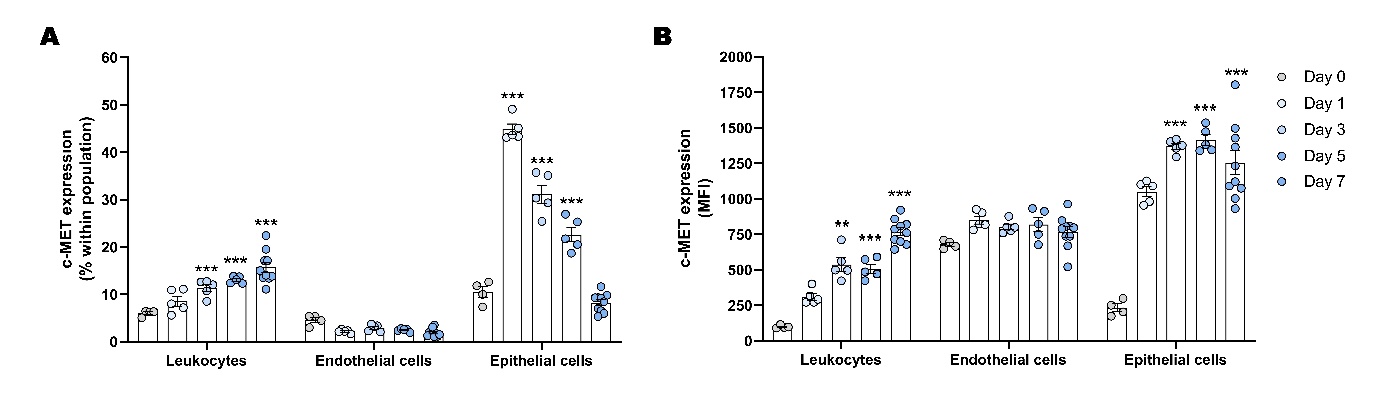


**Figure S3.** c-MET expression is enhanced in immune and epithelial cells during BLM-induced lung inflammation. (A) Percentage of c-MET^+^ and (B) MFI of c-MET among immune, endothelial and epithelial cells in the lung of vehicle-administered mice (day 0) and throughout the inflammatory phase of BLM-induced PF (day 1, day 3, day 5 and day 7). n = 4–10 *per* group. Data are expressed as mean ± SEM and were analyzed with one-way ANOVA with Tukey’s multiple comparisons test. **p* < 0.05, ***p* < 0.01, ****p* < 0.001, comparison with vehicle-administered mice.


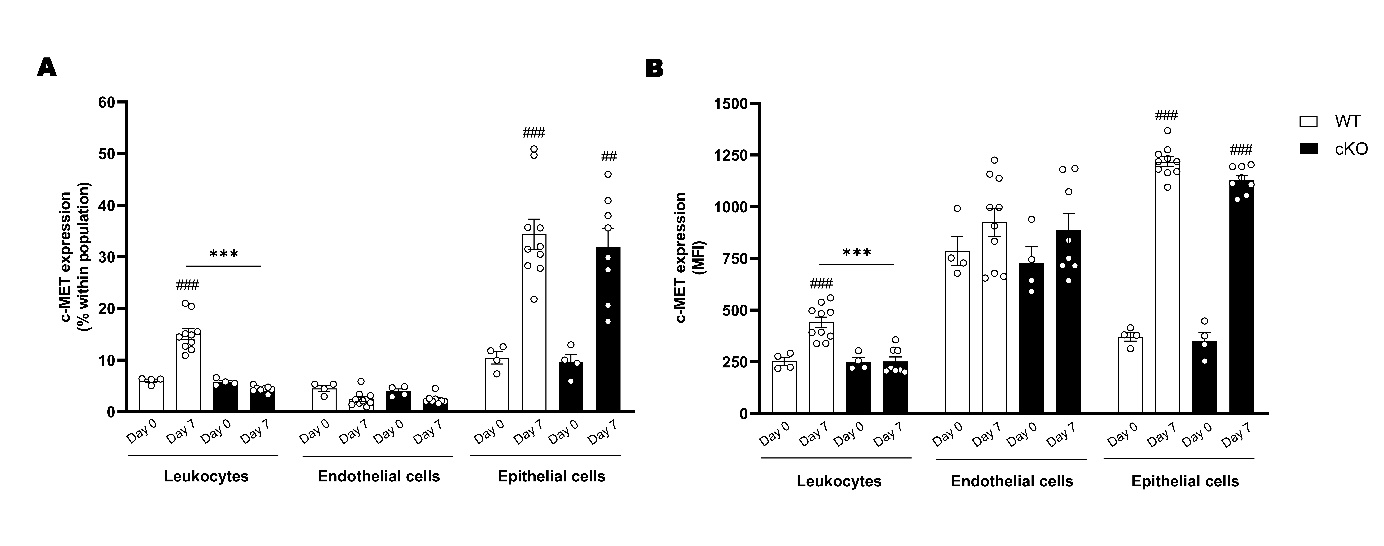


**Figure S4.** c-MET expression is effectively deleted in immune cells from *c-Met*-deleted cKO mice during BLM-induced lung inflammation. (A) Percentage of c-MET^+^ and (B) MFI of c-MET among immune, endothelial and epithelial cells in the lungs of WT and cKO mice, vehicle-administered (day 0) and 7 days after BLM administration. n = 4–10 *per* group. Data are expressed as mean ± SEM and were analyzed with one-way ANOVA with Tukey’s multiple comparisons test. ****p* < 0.001, comparison between genotypes. ##*p* < 0.01, ###*p* < 0.001, comparison with vehicle-administered mice.


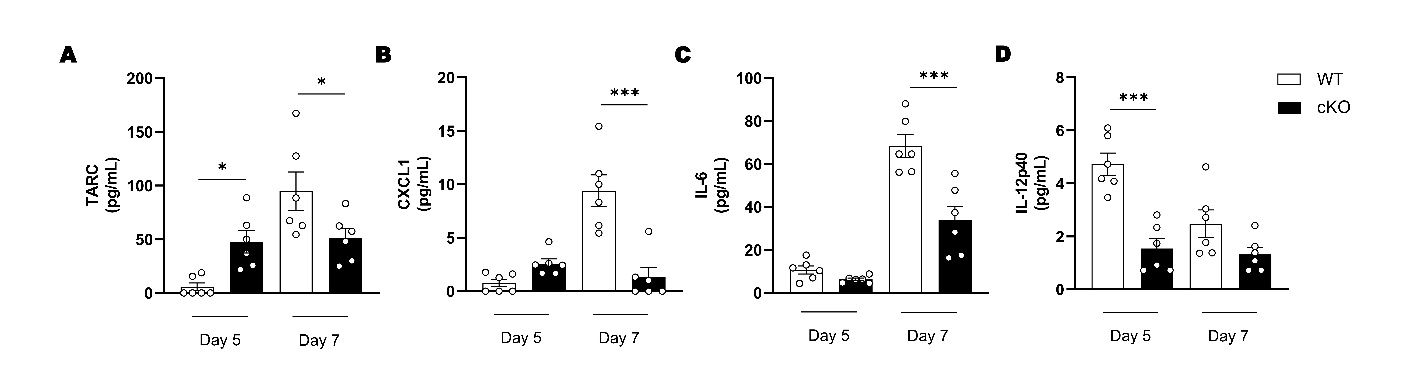


**Figure S5.** Levels of (A) TARC, (B) CXCL1, (C) IL-6 and (D) IL-12p40in BAL fluid from WT and cKO mice at day 5 and 7 after BLM administration. n = 6 *per* group. Data are expressed as mean ± SEM and were analyzed by Student’s two-tailed t test. **p* < 0.05, ***p* < 0.01, ****p* < 0.001.


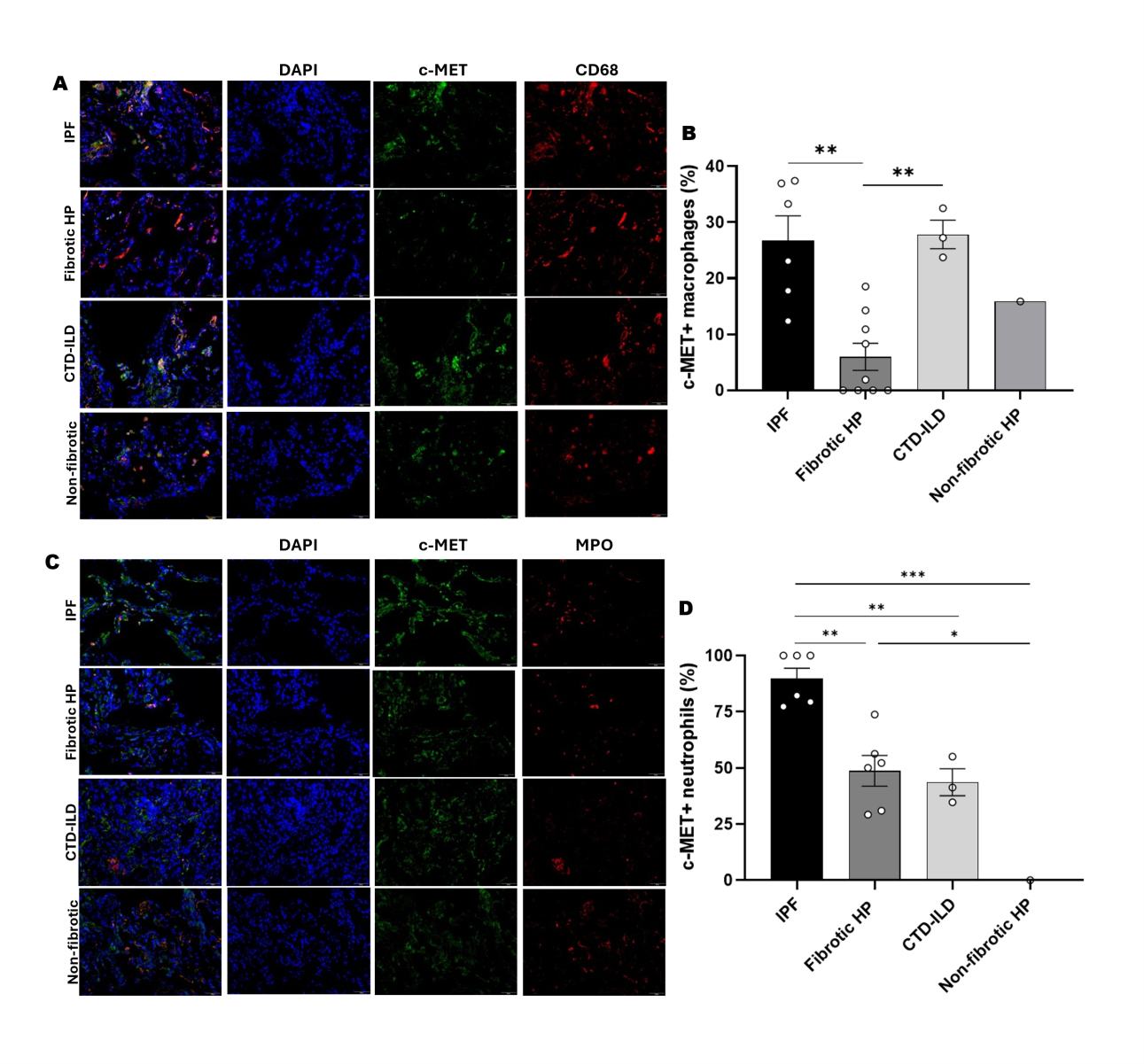


**Figure S6.** Profile of c-MET expression in neutrophils and macrophages among distinct interstitial lung diseases. (A) Representative images of immunofluorescence on lung sections from a cohort of patients confirms c-MET (green) in macrophages (red) and (D) respective expression quantification. (C) Representative images of immunofluorescence on lung sections from a cohort of patients confirms c-MET (green) in neutrophils (red) and (D) respective expression quantification. Original magnification ×20, scale bar 50 µm. n = 1–9 *per* group. Data are expressed as mean ± SEM and were analyzed with Kruskal-Wallis test with Dunn's multiple comparisons test.. **p* < 0.05, ***p* < 0.01, ****p* < 0.001.

**Figure S7.** Profile of c-MET expression in T cells, neutrophils and macrophages among distinct interstitial lung diseases. Heatmap of the c-MET expression profiles based on mean expression percentages in immune cell populations among distinct interstitial lung diseases.
